# Cognitive Bias Modification for Behavior Change in Alcohol and Smoking Addiction: Bayesian Meta-Analysis of Individual Participant Data

**DOI:** 10.1007/s11065-018-9386-4

**Published:** 2019-01-14

**Authors:** Marilisa Boffo, Oulmann Zerhouni, Quentin F. Gronau, Ruben J. J. van Beek, Kyriaki Nikolaou, Maarten Marsman, Reinout W. Wiers

**Affiliations:** 1 Department of Psychology, University of Amsterdam, Nieuwe Achtergracht 129B, 1018 Amsterdam, WS Netherlands; 2 Department of Psychology, University Paris Nanterre, Paris, France; 3 Trimbos Instituut, Netherlands Institute of Mental Health and Addiction, Utrecht, Netherlands; 4 School of Psychology, University of Sussex, Falmer, UK

**Keywords:** Alcohol, Tobacco, Smoking, Cognitive bias modification, Behavior change intervention, Meta-analysis, Bayesian meta-analysis

## Abstract

Cognitive Bias Modification (CBM) refers to a family of interventions targeting substance-related cognitive biases, which have been found to play a role in the maintenance of addictive behaviors. In this study, we conducted a Bayesian meta-analysis of individual patient data from studies investigating the effects of CBM as a behavior change intervention for the treatment of alcohol and tobacco use disorders, in individuals aware of the behavior change goal of the studies. Main outcomes included reduction in the targeted cognitive biases after the intervention and in substance use or relapse rate at the short-to-long term follow-up. Additional moderators, both at the study-level (type of addiction and CBM training) and at the participant-level (amount of completed training trials, severity of substance use), were progressively included in a series of hierarchical mixed-effects models. We included 14 studies involving 2435 participants. CBM appeared to have a small effect on cognitive bias (0.23, 95% credible interval = 0.06–0.41) and relapse rate (−0.27, 95% credible interval = −0.68 – 0.22), but not on reduction of substance use. Increased training practice showed a paradoxical moderation effect on relapse, with a relatively lower chance of relapse in the control condition with increased practice, compared to the training condition. All effects were associated with extremely wide 95% credible intervals, which indicate the absence of enough evidence in favor or against a reliable effect of CBM on cognitive bias and relapse rate in alcohol and tobacco use disorders. Besides the need for a larger body of evidence, research on the topic would benefit from a stronger adherence to the current methodological standards in randomized controlled trial design and the systematic investigation of shared protocols of CBM.

**Electronic supplementary material:**

The online version of this article (10.1007/s11065-018-9386-4) contains supplementary material, which is available to authorized users.

## Introduction

In the past decade, a new family of neurocognitive training paradigms, collectively called Cognitive Bias Modification (CBM), has received increasing attention as a potential low-threshold and easy-to-administer group of adjunct interventions for the treatment of addictive behaviors. CBM includes a variety of computerized training paradigms aimed at interfering with attentional, behavioral, or evaluative cognitive processes triggered by addiction-related cues in the environment. These *cognitive biases* have been found to play a role in maintaining addictive behaviors (for a review, see Wiers et al. 2013), leading researchers to the development of tools that could effectively modify these biases and, in turn, advance the treatment of addiction.

Typically, CBM training paradigms are based on the same methods used to assess the target cognitive bias, that is, speeded reaction-time tasks where participants have to react to disorder-relevant and control stimuli presented with some form of stimulus-response contingency (e.g., bias scores comparing responses for two task contingencies, such as responses toward or away from addiction-relevant stimuli). When the original assessment task is adapted for training, the built-in stimulus-response contingency is manipulated in order to create, through repeated practice, a new dominant stimulus-response association competing with, and counteracting, the existing dominant response toward the addiction-relevant cues. For example, in order to manipulate selective attention towards motivationally salient substance-related cues (i.e., attentional bias), researchers adjusted the Visual Probe Task (MacLeod et al. 1986). In this task, participants have to respond to a probe presented at the location of one of two stimuli displayed next to each other (or on top of each other) on the computer screen, such as a picture of a package of cigarettes and a smoking-unrelated picture. In the assessment version of the task, the probe is presented equally often at the location previously occupied by both types of stimuli. Typically, participants respond faster when the probe appears at the location on which their attention was already focused (Posner et al. 1980), that is, in the case of smokers, on the smoking-related stimulus. In the version of the task used to deliver Attentional Bias Modification (Field et al. 2007; MacLeod et al. 2002; Schoenmakers et al. 2007), the stimulus-response contingency is manipulated so as to systematically present the probe at the location of the neutral stimulus, thus training participants to consistently shift attention away from substance-related cues and to attend to neutral cues instead. The underlying idea is that repeated training can reduce or even invert the targeted biases, which in turn should lead to or help behavioral change (Wiers et al. 2013).

Similar contingencies have been introduced in other tasks used to assess different biases, such as the Approach Avoidance Task (Rinck and Becker 2007; Wiers et al. 2009), which is aimed at capturing approach action tendencies towards substance-related cues and has been modified to deliver Approach Bias Modification training (Wiers et al. 2010, 2011); the Go/No-Go task, which has been modified with the goal of inhibiting an instrumental response or behavioral approach towards reward-related cues (Selective Inhibition Training SIT , Houben et al. 2010a; for a meta-analysis, see Allom et al. 2016 and Jones et al. 2016) or Evaluative Conditioning training to change evaluative associations (Houben et al. 2010b, 2011; Zerhouni et al. 2018).

CBM training paradigms could be executed from the patient’s own home, potentially enhancing clinical outcomes at a minimum cost in terms of time and effort for both patients and health care professionals. The appeal of such computerized forms of cognitive training has led to both a proliferation of experimental research on the theoretical underpinnings of CBM and its short-lived effects, and to the first systematic evaluations of the therapeutic effects of CBM as a complementary clinical treatment aimed at behavior change. For a narrative review of the two classes of studies, see Wiers et al. (2018). The few meta-analyses conducted on the topic concluded that CBM has very small to no significant effects on the targeted bias(es), substance use, or symptoms of addiction (Cristea et al. 2016; Mogoaşe et al. 2014). However, both meta-analyses did not distinguish between the two qualitatively different classes of studies, and pooled the results in the same analyses. One class involves fundamental mechanism-oriented studies typically including participants not affected by substance use problems and not motivated to change their addictive behavior. The other class includes effectiveness studies and randomized controlled trials in clinical and subclinical populations.

Although from a pragmatic point of view pooling results across all CBM studies would provide a broad overview of the state of affairs of CBM as a research field, the meta-analytic blending of more fundamental and proof-of-principle studies and effectiveness studies in clinical and subclinical samples may lead to imprecise and misleading estimations about the clinical efficacy or effectiveness of CBM as a treatment method (Wiers et al. 2018). The former proof-of-principle studies have the primary goal of testing causal hypotheses on the relation between targeted cognitive biases and short-lived changes in behavior, typically with a taste-test, in participants not suffering from the target disorder (typically students), while the latter specifically target the population affected by the disorder of interest and are explicitly aimed at behavior change. Although fundamental research does and should provide evidence and suggestions for further clinical applications of behavior change principles, it is not meant nor designed to evaluate the therapeutic effects of such principles implemented into a behavior change treatment program (Sheeran et al. 2017; Wiers et al. 2018).

The goal of this meta-analysis was to quantify the existing evidence on the effectiveness of CBM for addictive behaviors as a *behavior change intervention*. Therefore, the meta-analysis exclusively focused on studies evaluating the clinical effects of any kind of CBM intervention targeting cognitive biases in problematic alcohol or tobacco use. Studies were included if designed with the explicit goal of inducing behavior change, excluding proof-of-principle studies in participants without the shared goal of behavior change. We only included behavior-change CBM studies in the alcohol and tobacco addiction domains since at the time of the initial literature search (May 2016) there was no published report of CBM intervention studies for other substances (e.g., cannabis, cocaine, or opiates), but solely cross-sectional assessment studies of different types of cognitive biases (e.g., Cousijn et al. 2011; Hester et al. 2006; Field et al. 2006; Lubman et al. 2000; Zhou et al. 2012).

Furthermore, conventional study-level systematic reviews often lack adequate power to detect clinically relevant predictors and moderators of treatment outcomes as effect size estimates are heavily dependent on the sample size of the individual clinical studies, which themselves can be underpowered. Therefore, this meta-analysis used individual patient data from the included studies instead of study-level aggregated estimates of effect sizes as to maximize the use of all available evidence to detect a true effect and explore study variability (e.g., type of targeted addiction, type of CBM intervention, intervention setting) and participants characteristics (severity of substance use problems and training adherence in terms of amount of completed training trials) as moderators of primary CBM outcomes (i.e., change in the target cognitive bias and substance use behavioral outcomes, such as reduction in substance use or relapse rate).

Although individual patient data meta-analyses are a powerful instrument for a more comprehensive analysis of all available evidence, providing deeper insight into the mechanisms underlying an effect, they are not to be taken lightly as they require a careful evaluation of the necessary workflow and expertise upfront. Individual patient data meta-analyses are more complex and challenging than conventional meta-analyses. These methods are more time and resource intensive since they are dependent on retrieving the individual patient data from researchers and on establishing an often long-lasting back-and-forth communication with researchers regarding the individual patient data and any missing or discrepant information found during the data checking. Indeed, it took around 1.5 years and an intense communication with the respective first authors in order to retrieve the raw datasets of the studies included in our meta-analysis, partially due to the very sensitive and confidential nature of clinical data. Further, individual patient data meta-analyses require making complex decisions about data handling in order to ensure the accuracy of outcomes (e.g., how to harmonize different outcome measures across studies or how to handle missing data) and advanced statistical expertise due to the complexity of the hierarchical modeling involved.

Our meta-analytic approach involved testing a series of multilevel mixed-effects models including relevant study- and participant-level moderators on the pooled individual patient data from all studies. All models were tested within the Bayesian statistical framework in order to benefit from the advantage of quantifying the available evidence in favor or against a hypothesized effect, outweighing some of the limitations of the classic frequentist approach. Statistical inference in the frequentist approach typically relies on a *p*-value, that is, the conditional probability that the observed data (i.e., *p*(data | hypothesis), H_0_) – or more extreme data – may be observed under the assumption that the null hypothesis of a zero effect, is true. While this is the favored standard approach in behavioral and cognitive science, it suffers from several limitations.

First, the *p*-value does not provide information on the probability of the tested hypothesis H_1_ and hence does not allow for a direct corroboration of the hypothesis of interest. In the frequentist approach, it is not possible to estimate the probability of the hypothesis being true given the data, or *p*(hypothesis H_1_ | data). Because *p*(hypothesis H_1_ | data) can be weakly correlated to *p*(data | hypothesis H_1_), (*r* = .38, Krueger 2001; Krueger and Heck 2017), the *p*-value does not allow us to directly draw any inference about the hypothesis. The casual inference from *p*(data | hypothesis H_1_) to *p*(hypothesis H_1_ | data) has therefore little justification. Second*,* the *p*-value has been shown to have a high sampling variation, depending on effect size, sampling variability, and sample size (Murdoch et al. 2008; Cumming 2014). Third, as samples become very large, even small deviations from the null hypothesis have a higher probability of passing the significance threshold, since an increase in sample size decreases standard error (Kruschke 2013; Kruschke and Lidell 2017).

In contrast to classical methods, which cannot distinguish between the absence of evidence (i.e., the data are uninformative) and the evidence of absence (i.e., the data support the null hypothesis H_0_), Bayesian methods allow modeling and quantification of the evidence for each hypothesis, rather than relying on a dichotomous decision (Wagenmakers et al. 2018). This is why we conducted the meta-analysis within the Bayesian framework, using a hierarchical random-effects modeling approach similar to that of Marsman et al. (2017). In this approach, information about the effect can be estimated from the individual participants nested within individual studies – similar to the frequentist approach – but yielding posterior distributions for the effect sizes. Individual effect sizes from each study are therefore not considered alone, rather, they are assumed to be drawn from a group-level normal distribution of effect sizes (i.e., the group-level model), whose variance and mean reflects heterogeneity between studies and the mean effect size in the group of studies. Further, the hierarchical structure of the group-level model shrinks individual results that are uncertain and relatively extreme to the group mean effect (Efron and Morris 1977; Lee and Wagenmakers 2014), and it is generally possible to obtain narrower posterior credible intervals since the uncertainty of the individual study effects are reduced by the information borrowed from other statistically similar studies. Finally, Bayesian hypothesis testing allows us to explicitly quantify the evidence in favor of the null hypothesis versus a specific alternative hypothesis (i.e., Bayes factor; Marsman et al. 2017; Wagenmakers et al. 2018).

Although providing a compelling framework to test alternative hypotheses, Bayesian methods also suffer from important downsides. It could also be argued that the possibility of including prior information is an advantage (e.g., Vanpaemel 2010), however, a common criticism is that the Bayes factor is sensitive to the choice of the prior distribution of model parameters, consequently influencing the statistical inference regarding the plausibility of a certain model over another. We therefore evaluated the robustness of the conclusions across three different prior distributions. Another, more pragmatic, downside of utilizing a Bayesian approach includes the difficulties in the specification of the model(s) to be tested since it is not possible, nor feasible to test all possible models included in a parameter space, and the need for very high computational power with standard software.

The present study reported the results of a Bayesian meta-analysis of behavior change studies evaluating the effectiveness of any kind of CBM intervention with participants suffering from alcohol and tobacco use disorders or problems. The main goal was to establish a) whether CBM interventions impact the targeted cognitive bias(es) and substance use outcomes (i.e., reduction in substance use and relapse rate), and b) if relevant study- and participant-level characteristics moderate these effects. Characteristics examined included targeted substance use disorder, type of CBM training deployed in the intervention, intervention setting, amount of completed training trials, and severity of substance use problems.

## Methods

### Study Eligibility Criteria

Studies were included if they met the following eligibility criteria: (1) were published in English, (2) included a CBM intervention directed at alcohol or tobacco use, for example, Approach Bias Modification (e.g., Wiers et al. 2010, 2011), Attentional Bias Modification (e.g., Field et al. 2007; Schoenmakers et al. 2007), Evaluative Conditioning or Selective Inhibition Training (e.g., Houben et al. 2010a, b, 2011);, (3) included outcome measures of cognitive bias, substance use, or relapse rate;, (4) participants were randomly allocated to intervention conditions, (5) included a comparison between a control condition (active or inactive) and a CBM intervention (studies that featured multiple control conditions or interventions were also included), and (6) participants were aware that the goal of the study was behavior change (e.g., abstinence, reduction of substance intake or of addiction problems). The latter criterion was added to distinguish clinical effectiveness and behavior change CBM studies from more fundamental proof-of-principle lab-studies (Sheeran et al. 2017), which are aimed at experimentally manipulating target psychological processes in order to establish causality, and not to evaluate the effects of an intervention for a particular condition (for a more extensive discussion on this distinction in the CBM field, see Wiers et al. 2018).

### Study Identification and Selection Process

The meta-analysis was performed in compliance with the Preferred Reporting Items for Systematic Review and Meta- analysis (PRISMA) individual patient data Statement (Stewart et al. 2015). PsychINFO, Medline, Web of Science, Embase, and the Cochrane Library bibliographic databases were systematically searched from inception to May 18, 2016. Three sets of keywords were used covering the constructs of interest, namely, cognitive bias, addiction, and study type. For the cognitive bias set the main keywords were “cognitive bias”, “attentional bias”, “approach bias”, “response inhibition”. The second group of keywords related to addiction included “alcohol”, “drinking” and “tobacco”. The third group of keywords covering intervention types consisted of “longitudinal”, “(re)training”, “intervention” and “task”. All sets of keywords and subject headings for all databases were compiled with the support of the health librarian of the University of Amsterdam. They were generated both from a set of relevant keywords compiled by two CBM researchers and using a reference set of articles known to the authors as meeting the inclusion criteria. For all sets, additional keywords were used based on the bibliographic categorization of relevant papers reporting behavior-change CBM studies. The full list of search strings and results per bibliographic database is reported in the Supplementary Material. The references of the included studies were also searched systematically for missed studies. Two of the authors independently examined titles and abstracts of 2579 search results and further screened the full text of potential studies for full eligibility. In case of unclear or missing information to decide upon inclusion, the first authors of the candidate studies were contacted. Criterion 6 was evaluated by screening the full-texts for information regarding whether participants were fully informed about the behavior change goal of the study (i.e., that they would receive a treatment intervention). When unclear or no available information was given, first authors were explicitly asked to clarify what information was provided to the participants about the study goals. In case of disagreement regarding inclusion, consensus was sought through discussion with other two members of the team expert in CBM and addiction clinical research (MB and KN).

### Data Collection and Data Items

Authors of eligible articles were contacted for permission to use their raw data sets. They were asked to provide individual raw data on demographic (age and gender), clinical (severity of substance use problems), and intervention characteristics, including information regarding randomized group, baseline, post-intervention, and follow-up total scores of outcomes (cognitive bias(es), substance use, relapse), and training adherence information (total number of training sessions completed * amount of training trials per session). Integrity of the data included in the collected datasets was screened against data reported in the published reports and when discrepancies were found, the first authors were contacted for clarifications.

We also coded study-level variables, which were available from the full reports, including, targeted addiction, type of CBM intervention, type of control condition (active or inactive), intervention setting (supervised, such as lab or clinic, or unsupervised, such as online), assessment time points, and types of outcomes measures for cognitive bias, substance use, and relapse rate.

The analyses were conducted separately for each outcome, that is, cognitive bias, reduction in substance use, and relapse rate. The selection of moderator variables, and interactions between moderators, has been based on literature related to moderators of CBM training effects (i.e., severity of substance use problems; Eberl et al. 2013; intervention setting, Price et al. 2016) and hypotheses regarding intervention parameters that can impact effectiveness (i.e., type of CBM training and training adherence in terms of amount of completed training trials).

### Risk of Bias Assessment in Individual Studies

We examined the risk of bias in the included studies using the criteria of the Cochrane Collaboration risk of bias assessment tool (Higgins et al. 2011). Two of the authors independently evaluated the included studies to determine whether there was a risk for bias related to selection, performance, detection (for cognitive bias and behavioral outcomes), attrition (for cognitive bias and behavioral outcomes), and reporting. In case of unclear risk of bias for one or more key domains, the first authors of the included studies were contacted for clarifications.

### Individual Patient Data Meta-Analysis

Included studies used a mixture of measures to assess the study outcomes, that is, reaction-time tasks for the targeted cognitive bias(es) and self-report measures of substance use during a defined time frame (see Table 1). Cognitive biases are always assessed with reaction-time tasks involving the presentation of substance-related cues (typically pictures) across trials involving a stimulus-response manipulation, for example, alcohol-related and non-alcohol-related stimuli are presented in both approach and avoid trial formats in the Approach Avoidance Task in order to assess approach bias towards the alcohol-related relative to the non-alcohol-related cues. Another example includes the presentation of a probe appearing at the location of either the alcohol-related or the non-alcohol-related stimulus in the Visual Probe Task in order to assess attentional bias. A summary score is typically obtained by computing the relative difference in mean (or median) response times (RT) to substance-related stimuli presented in the different trial conditions (e.g., [alcohol/avoid – alcohol/approach] – [non-alcohol/avoid – non-alcohol/approach] for approach bias, or probe/non-alcohol – probe/alcohol for attentional bias). Such scores index the strength of cognitive bias towards substance-related stimuli relative to control stimuli (e.g., the difference in RT between approaching rather than avoiding alcohol-related cues and approaching rather than avoiding non-alcohol-related cues). The scoring logic is the same across the different paradigms belonging to this family of neuropsychological tasks. Therefore, cognitive bias scores were standardized within each study by transforming them into z scores, which has the added benefit of removing any confounder related to using, for example, slightly different task data cleaning procedures, or using mean or median RT scores.

**Table 1:** Characteristics of included studies: study ID, type of addiction and sample, age and gender, type of CBM intervention, control condition, training schedule, task stimuli, assessment of outcomes (measures and time points), training setting, completer rate

Study	Type of addiction (N)	Age / gender	CBM intervention	Control condition	Training schedule	Task stimuli	Cognitive Bias assessment	Substance use assessment	Relapse assessment	Setting	Completer rate
Begh et al. 2015	Tobacco (N = 118^a^ adult smokers, recruited in stop smoking services and medical general practices)	Mean age = 44.8, SD = 12.7; 69 (58%) females	AtBM + TAU (smoking cessation program); VPT, probe replaced neutral pictures (100%) *n* = 60	Active: sham AtBM training + TAU (smoking cessation program); VPT, probe replaced smoking or neutral pictures (50%) *n* = 58	1 session x week for 5 weeks 192 training trials x session	18 matched pairs of smoking-related pictures (smoking related objects and people smoking cigarettes) and control pictures (everyday objects, such as staplers or keys, and people engaged in everyday activities, such as using the phone)	Measures: visual Stroop task, VPT Time points: baseline, 1, 5, 9, and 10 weeks after end of training	_	Measures: electronic diary, exhaled carbon monoxide Time points: baseline, each training session, 1, 5, 9 and 10 weeks after end of training	Supervised in smoking clinic	73% at 1 week, 75% at 5 weeks, 69% at 9 weeks, and 64% at 10 weeks after end of training
Clerkin et al. 2016	Alcohol (*N* = 86 community-recruited adult heavy drinkers with elevated social anxiety symptoms)	Mean age = 44.3, SD = 10.9; 35 (41%) females	AtBM + real or sham social AtBM training for social anxiety (factorial design); VPT, probe replaced neutral pictures (100%) + positive feedback on response accuracy and RT *n* = 42	Active: sham AtBM training + real or sham AtBM training for social anxiety; VPT, probe replaced alcohol or neutral pictures (50%) *n* = 44	2 sessions x week for 4 weeks (max 6 weeks) 144 training trials x session	60 pairs of matched alcohol-related pictures (alcoholic drinks) and neutral pictures (non-alcoholic drinks, including water and other drinks). All pictures include both passive (beverage only) and active (person drinking) contexts	Measures: VPT Time points: baseline, 1 and 4 weeks after end of training	Measures: Daily Drinking Questionnaire (total amount of drinks in the past week) Time points: baseline, 1 and 4 weeks after end of training	_	Supervised in research lab	81% at 1 and 4 weeks after end of training
Cox et al. 2015	Alcohol (*N* = 148 community-recruited, adult at-risk drinkers)	Mean age = 28.8, SD = 14.4; 140 (57%) females	AtBM with or without group motivational workshops (LEAP; factorial design); AACTP paradigm *n* = 77	Inactive: no training with or without LEAP)	1 session x week for 4 weeks 500 training trials x session	Alcohol-related pictures (bottles of alcoholic drinks) Neutral pictures (bottles of soft-drinks)	–	Measures: Drinking Record Questionnaire (quantity and frequency of drinking in past 12 weeks; mean weekly drinking index) Time points: baseline, immediately and 12 and 24 weeks after end of training	–	Supervised in research lab	79% at post-training, 59% at 12 weeks and 47% at 24 weeks after end of training
Eberl et al. 2013	Alcohol (N = 475^b^ alcohol dependent adult inpatients)	Mean age = 46, SD = 9; 118 (25%) females	ApBM + TAU (CBT-based residential treatment); AAT, alcohol stimuli presented in push format (100%) *n* = 248	Inactive: no training + TAU (CBT-based treatment) *n* = 227	2 sessions x week for 6 weeks 200 training trials x session	20 alcohol-related pictures (familiar common alcoholic drinks) 20 neutral pictures (familiar common soft-drinks)	Measures: AAT, VPT Time points: baseline, start of each training session, end of training	–	Measures: Self-reported abstinence Time points: 12 months after discharge	Supervised in residential clinic	AAT: 71% at baseline, 72% at post-training. Relapse: 75% at 12 months after discharge
Elfeddali et al. 2016	Tobacco (*N* = 434, online-recruited adult smokers, actively confirming a quit-attempt after baseline)	Mean age = 40.8, SD = 11; 299 (69%) females	AtBM; VPT, probe replaced neutral pictures (100%) *n* = 224	Active: sham AtBM training; VPT, probe replaced smoking or neutral pictures (50%) *n* = 210	2 sessions x week for 3 weeks (max 4 weeks) 240 training trials x session	96 matched pairs of smoking-related pictures (e.g., cigarette packs, smoking people) and neutral pictures (e.g., pencil packages, non-smoking people)	Measures: VPT, AAT Time points: baseline, immediately after end of training, and 6 months after baseline.	–	Measures: self-reported smoking abstinence Time points: 6 months after baseline	Unsupervised, online intervention	48% at post-training, 31% at 6 months after baseline
Kong et al. 2015	Tobacco (N = 60 adolescent smokers recruited in high schools in the US and NL)	Mean age = 17, SD = 1.2; 21 (35%) females	ApBM + TAU (CBT for smoking cessation); AAT, smoking stimuli presented in push format (100%) *n* = 29	Active: sham ApBM training + TAU (CBT for smoking cessation); AAT, smoking and control stimuli presented in push and pull format equally often *n* = 31	1 session x week for 4 weeks 300 training trials x session	20 smoking-related pictures (e.g., cigarettes, cigarette packs, adolescents smoking) 20 neutral pictures (e.g., pencil, lipsticks)	Measures: AAT Time points: baseline and 12 weeks after end of treatment	–	Measures: 7-days self-reported smoking abstinence verified through measure of cotinine levels. Time points: immediately and 12 weeks after end of treatment.	Supervised in schools (individual sessions)	32% at end of treatment and 58% at 12 weeks after end of treatment
Lopes et al. 2014	Tobacco (*N* = 67 adult smokers recruited from university staff and students enrolled in smoking cessation program)	Mean age 45.1; SD = 12.2; 42 (63%) females	1. 3 sessions AtBM + TAU (group CBT for smoking cessation); probe replaced neutral pictures (100%) n = 22 2. 1 session AtBM +2 sessions sham training + TAU VPT, probe replaced neutral pictures (100%) *n* = 22	Active: sham AtBM training + TAU (group CBT for smoking cessation); 1 session VPT, probe replaced smoking or neutral pictures (50%), with different set of smoking stimuli; 2 sessions VPT with neutral stimuli *n* = 23	3 sessions over first 2 weeks of TAU 576 training trials per session	48 pairs of matched smoking-related (e.g., cigarette packs, lighter) and neutral pictures (e.g., wallet, cell phone) + 24 pairs of neutral pictures taken from the IAPS database for the control condition	Measures: VPT (50, 500, 2000 ms SOAs)^c^ Time points: baseline, 24 h, 4 weeks, 6 and 12 months after the end of training	Measures: self-reported number of cigarettes smoked/day, exhaled carbon monoxide Time points: baseline, 24 h, 4 weeks, 6 and 12 months after the end of training	Measures: self-reported continued smoking Time points: baseline, 24 h, 4 weeks, 6 and 12 months after the end of training	Supervised in research lab	100% at 24 h, 75% at 1, 6 and 12 months after end of training
Machulska et al. 2016	Tobacco (N = 139^d^ adult smokers recruited in inpatient rehab clinic)	Mean age = 42.7, SD = 10.4; 37 (27%) females	ApBM + TAU (3 sessions motivational interviewing and psychoeducation); AAT, smoking stimuli presented in push format (100%) *n* = 73	Active: sham ApBM training + TAU (3 sessions motivational interviewing and psychoeducation; AAT, smoking and control stimuli presented in push and pull format equally often *n* = 66	1 session x day for 4 Days 250 training trials x session	15 smoking-related pictures depicting models at stages of smoking rituals (e.g., taking a cigarette our of the pack, lighting a cigarette, killing a cigarette) 15 neutral pictures depicting models at different stages of tooth brushing (e.g., preparing toothbrush with toothpaste, putting toothbrush in the mouth)	Measures: AAT Time points: baseline, immediately after end of training	Measures: self-reported number of cigarettes/day Time points: baseline and 12 weeks after end of training	–	Supervised in clinic	76% immediately after the end of training. No attrition data available for the 12-week follow-up
McHugh et al. 2010	Tobacco (*N* = 50 community-recruited adult smokers)^e^	Mean age = 37.9, SD = 13.8; 18 (35%) females	AtBM; VPT, probe replaced neutral pictures in most of the trials (85%)	Active: sham AtBM training; VPT, probe replaced smoking or neutral pictures (50%)	1 session 560 training trials	20 pairs of matched smoking-related pictures (smoking-related scenes, such as woman holding cigarette to mouth, cigarette beside ashtray) and neutral pictures (e.g., e.g. woman applying lipstick, pen beside bowl)	Measures: VPT Time points: baseline, immediately after end of training	–	–	Supervised in research lab	100% at end of training.
Schoenmakers et al. 2010	Alcohol (n = 43 alcohol-dependentadult in- and outpatients)	Mean age = 45, SD = 9.8; 10 (23%) females	AtBM + TAU (CBT); VPT, probe replaced neutral pictures (100%) + positive feedback on response accuracy and RT *n* = 21	Active: placebo training + TAU (CBT); categorization task, same stimuli used in AtBM training n = 22	5 sessions over 3 weeks (2 x week) 528 training trials x session	60 pairs of matched alcohol-related pictures (e.g., alcohol drinks and objects) and neutral pictures (e.g., soft-drinks, furniture and stationary)	Measures: VPT (trained and untrained alcohol stimuli at post-training) Time points: baseline, 3–4 days after end of training	–	Measures: self-reported abstinence confirmed by medical record Time points: 12 weeks after end of training	Supervised in clinic	86% immediately and 81% at 12 weeks after end of training
Wiers et al. 2015a, b	Alcohol (N = 32^f^ alcohol dependent adult inpatients)	Mean age = 44, SD = 7.6; 32 (100%) males	ApBM + TAU (CBT-based residential treatment); AAT, alcohol stimuli presented in push format (90%) and neutral stimuli in pull format (90%) *n* = 15	Active: sham ApBM training; AAT, alcohol and control stimuli presented in push and pull format equally often *n* = 17	2 x week for 3 weeks 400 training trials x session	40 alcohol-related pictures (familiar common alcoholic drinks) 40 neutral pictures (familiar common soft-drinks)	Measures: AAT Time points: baseline and immediately after end of training	–	Measures self-reported abstinence (not part of the final report) Time points: 12 months after discharge from the clinic	Supervised in clinic	AAT: 100% after end of treatment Relapse: 84% after 12 months from discharge from the clinic
Wiers et al. 2011	Alcohol (*N* = 214 alcohol dependent adult inpatients)	Mean age = 45.3, SD = 8; 52 (24%) females	1. ApBM + TAU (CBT-based residential treatment); AAT, alcohol stimuli presented in push format (100%); explicit instructions to react to alcohol and non-alcohol stimuli *n* = 56 2. ApBM + TAU; AAT, alcohol stimuli presented in push format (100%); implicit instructions to react to stimulus format n = 52^g^	1. Active: sham ApBM training + TAU (CBT-based residential treatment); AAT, alcohol and control stimuli presented in push and pull format equally often *n* = 55 2. Inactive: waiting list + TAU n = 51^g^	1 x day for 4 days 200 training trials x session	20 alcohol-related pictures (familiar common alcoholic drinks) 20 neutral pictures (familiar common soft-drinks)	Measures: AAT (trained and untrained stimuli at post-training), approach-avoidance IAT Time points: baseline, 1 weeks after end of training	–	Measures: self-reported abstinence Time points: 12 months after discharge from the clinic	Supervised in clinic	AAT: 86% at baseline (11 participants excluded for excessive error rate); 89% at 1 week after end of training (6 participants excluded for excessive error rate) IAT: 91% baseline and 93% 1 week after end of training Relapse: 87% at 12 months after discharge
Wiers et al. 2015a, b	Alcohol (*N* = 615 online recruited adult problem drinkers; data available for 312 participants (51%) who initiated the baseline)	Mean age = 45.9, SD = 11.2; 144 (46%) females^h^	1. AtBM; AACTP paradigm n = 56^i^ 2. ApBM; AAT, alcohol stimuli presented in push format (100%); explicit instructions to react to alcohol and non-alcohol stimuli *n* = 57 3. ApBM; AAT, alcohol stimuli presented in push format (100%); implicit instructions to react to stimulus format *n* = 67 4. ApBM; AAT, alcohol stimuli presented in push format (90%); implicit instructions to react to stimulus format n = 77^l^	1. Active: sham ApBM training; AAT alcohol and control stimuli presented in push and pull format equally often n = 55	4 sessions (max 14-day interval between sessions) AACTP: 200 training trials per session AAT: 220 training trials x session	Alcohol-related pictures (alcoholic drinks) and neutral pictures (non-alcoholic drinks, such as soft-drinks and water)	Measures: SRC Time points: baseline, 2 weeks after end of training	Measures: Time Line Follow Back (mean weekly drinking in the past two weeks) Time points: baseline, 2, 6 and 13 weeks after end of training	–	Unsupervised, online intervention	Completers: 22% 2 weeks, 18% 6 weeks, and 14% 13 weeks after the end of training
Wittekind et al. 2015	Tobacco (*N* = 257 adult heavy smokers recruited in online smoking-related forums)	Mean age = 43.2, SD = 11; 157 (61%) females	1. ApBM; AAT, alcohol stimuli presented in push format (100%); *n* = 87 2. ApBM; AAT, alcohol stimuli presented in push format (100%) + feedback on RT n = 85^l^	Inactive: waiting list *n* = 85	1 session 100 training trials	10 pairs of matched smoking-related pictures (e.g., smoking packs, smoking scenes) and neutral pictures (everyday scenes and objects)	–	Measures: self-reported amount of cigarettes/day Time points: baseline, 4 weeks after baseline	–	Unsupervised, online intervention	61% at four weeks after baseline

^a^Randomized participants were originally 119, one participant died shortly after enrolment

^b^Included participants were originally 499; 13 patients were excluded due to technical problems with the computer program and 11 patients dropped out at or after the baseline assessment (unclear if before or after being randomized)

^c^For the sake of comparability with the other included studies, only VPT scores computed on trials with 500 ms SOA were included in the meta-analysis

^d^Randomized participants were originally 186, but 41 participants withdrew during or immediately after the first session (22% of the original sample) and 6 participants were excluded due to excessive error rate in the assessment task

^e^Original sample included 64 participants; 13 excluded due to excessive error rate, 1 withdrew from the study

^f^The study was not originally designed to test clinical effects, rather neural effects of CBM. Therefore, participants who did not complete all study sessions were excluded (4 extra patients)

^g^Since the ApBM variants solely differed in procedural features and not in content, the two ApBM conditions were collapsed together and contrasted against the two control groups merged together, similarly to the original study

^h^Demographics were available only for the 314 participants who initiated the baseline assessment session

^i^Original group size was 58, two participants were excluded from the sample since they did not complete any AtBM training session

^l^The ApBM conditions were collapsed together for the meta-analyses (the ApBM variants differed in procedural features and not in content)

AACTP: alcohol attention-control training programme; AAT: approach avoidance task; AtBM: attentional bias modification; ApBM: approach bias modification; CBT: cognitive behavioral therapy; IAT: implicit association test; LEAP: life enhancement and advancement program; RT: reaction time; SD: Standard Deviation; SOA: stimulus onset asynchrony; SRC: Stimulus Response Compatibility Task; TAU: treatment as usual; VPT: visual probe task

Given that the same assessment task is normally recast for training, some studies included the evaluation of training effects on the targeted bias, or on a different type of cognitive bias, by also assessing it with an additional, different task paradigm (i.e., Begh et al. 2015; Eberl et al. 2013; Schoenmakers et al. 2010; Wiers et al. 2011). This approach has the advantage of detecting whether training effects generalize beyond the same task paradigm, that is, “far generalization”, which is different from “close generalization”, referring to an effect on untrained stimuli in the same task used for training (Wiers et al. 2013). Hence, when different measures of cognitive biases were used, all were included in the individual patient data analysis as a separate comparison.

The same z-score transformation was applied to measures of substance use, all of which consisted of similar retrospective, calendar-based measures of consumption during a defined time window (e.g., the Time Line Follow Back or questionnaires assessing quantity and/or frequency of substance use over a defined time window, usually one or two weeks; see Table 1). Hence, there was no substantial difference in the type of measure of substance use across studies preventing the application of a z-score transformation. Before standardizing the substance use measures, they were adjusted so as to index the average quantity of substance consumption per week, allowing for a direct comparison of training effects across the studies. Therefore, when weekly scores were not directly available, individual measures of tobacco use (i.e., number of cigarettes per day) were multiplied by seven in order to align them to the time window typically used in alcohol studies (i.e., amount of alcohol units or drinks per week). To do so, we also had to adjust one alcohol study by multiplying the alcohol-use outcome (i.e., mean number of drinks per day) by seven (Wiers et al. 2015b).

One of the moderators, severity of substance use problems, was also assessed differently across studies. Studies in the tobacco domain all used the same instrument to assess severity of smoking addiction (Fagerström Test for Nicotine Dependence, FTND; Heatherton et al. 1991). However, while most studies targeting alcohol addiction assessed severity with the Alcohol Use Disorder Identification Test (AUDIT; Saunders et al. 1993), two studies (Clerkin et al. 2016; Cox et al. 2015) used two other self-report measures, the Drinker Inventory of Consequences (Miller et al. 1995) and the Short Index of Problems (Feinn et al. 2003), respectively, with the latter being a short version of the former, therefore highly related to each other. These two measures of alcohol problems have shown to be moderately-to-highly associated with the AUDIT, supporting the idea that they conceptually measure similar constructs (Donovan et al. 2006). Therefore, they were also included in the moderation analyses for severity of substance use. Before conducting the analyses, all measures of severity of substance use were standardized by transformation into z scores across studies using the same measure (four for the AUDIT, one for the Drinker Inventory of Consequences and one for Short Index of Problems, seven for the FTND; one study did not include individual patient data on severity of substance use problems). All other moderator variables were standardized within each study before running the analyses.

Most of the studies included multiple assessment time points of both cognitive bias and substance use outcomes. However, to minimize the spread of difference in the follow-up duration, for all studies we included the first cognitive bias measurement available after the conclusion of the training. For substance use and relapse rate we included the measurements taken at the longest follow-up time point available. A duration-of-follow-up variable measured in weeks from the end of training was extracted from the study reports or the datasets including individual patient data, standardized within each study, and added in the main analyses as a covariate. Note that for one study the duration of the longest follow-up was slightly different across participants (range = 19–26 weeks; Elfeddali et al. 2016), therefore for this study we computed the mean duration of follow-up across participants (mean = 24).

For all studies, we contrasted the CBM intervention with the control condition. One study included two control conditions (Wiers et al. 2011), which were collapsed to avoid the exclusion of a substantial amount of observations, and were reported not to be different on any of the outcomes. If a study contained multiple CBM conditions or multiple measures for the same outcome, all conditions and outcomes were included in the analysis as separate comparisons. Note that two studies tested the combination of a CBM intervention with a different intervention with a factorial experimental design (Clerkin et al. 2016; Cox et al. 2015). For these studies, the relevant CBM training and control groups were collapsed over the other intervention levels. We also collapsed together the different Approach Bias Modification training conditions in Wiers et al. (2011), Wiers et al. 2015b) and Wittekind et al. (2015), since the training varieties administered to the groups only differed slightly, with no substantial difference in training effects in intention-to-treat analyses.^1^ Finally, due to the original study design comparing multiple CBM trainings against the same control condition, the Attentional Bias Modification and Approach Bias Modification training conditions in Wiers et al. (2015a, b) were each contrasted against the same control group in the analyses.

Missing outcome data was not estimated with imputation methods when the authors did not use one, that is, imputed data were used only when available in the original raw datasets. Note that, when including non-completers in the analyses, different missing data imputation methods were used across the studies, including single (i.e., last observation carried forward; Cox et al. 2015; Machulska et al. 2016; Schoenmakers et al. 2010) and multiple imputation (Wiers et al. 2015b), and the use of statistical methods robust to missing data, such as multilevel mixed models (Begh et al. 2015; Clerkin et al. 2016).

For each outcome, a one-stage individual patient data meta-analysis was conducted and all individual raw data sets were combined into a merged data set, with participants nested within studies. A series of meta-regression analyses was conducted on each of the three outcomes (*n* = 18 comparisons for cognitive bias, *n* = 7 comparisons for reduction in substance use and *n* = 8 comparisons for relapse), testing seven hierarchically organized models of increasing complexity against a base model (*M*_0_), which included only the main effect of training condition (i.e., training or control). The goal was to test whether study-level (duration of the follow-up measurement, type of CBM training and addiction disorder) and available participant-level (severity substance use problems and number of completed training trials)^2^ characteristics moderated the effects of CBM on the considered outcomes.

1. *M*_0_: training condition (i.e., training or control)
2. *M*_1_: *M*_0_ + duration of follow-up covariate
3. *M*_2_: *M*_1_+ addiction disorder (i.e., alcohol or tobacco)
4. *M*_3_: *M*_2_+ type of CBM training (i.e., Attentional Bias Modification or Approach Bias Modification)
5. *M*_4_: *M*_3_+ addiction disorder * type of CBM training
6. *M*_5_: *M*_4_+ No. of completed training trials
7. *M*_6_: *M*_5_+ No. of completed training trials * training condition
8. *M*_7_: *M*_6_+ severity of substance use problems

Control and training condition were coded −0.5 and + 0.5, respectively. Alcohol use disorder and Attentional Bias Modification training were coded −1 and tobacco use disorder and Approach Bias Modification training +1.

### Bayesian Individual Patient Data Meta-Analysis

The Bayesian analyses comprised two steps: (a) estimating the posterior distributions of the model parameters, and (b) computing the Bayes factor to compare a model against the baseline model *M*_0_.

In Bayesian parameter estimation, observed data are used to update knowledge about the model parameters (Wagenmakers et al. 2018). To this aim, we need to specify our knowledge about the model parameters before the data are observed by introducing a prior distribution that expresses prior knowledge or the relative plausibility of the possible values of the parameters. The information in the data is then used to update this prior distribution to a posterior distribution, which expresses our uncertainty about the unknown parameters after the data have been observed. The posterior distributions for the parameters of our models were estimated with R (R Core Team 2017) using the *rstan* package (Stan Development Team 2017). To summarize these posterior distributions, we report posterior means to indicate the strength of an effect, and 95% central credible intervals to indicate the uncertainty that is associated with the effect. If such an interval ranges between two values *a* and *b*, we can be 95% confident that the true value of the parameter lies between these values.

To compare the predictive accuracy of different models we use Bayes factors (e.g., Etz and Wagenmakers 2017). A Bayes factor for comparing *M*_1_ against *M*_0_, say, is expressed as

$$ {\mathrm{BF}}_{10}=\frac{p\left(\mathrm{data}|{M}_1\right)}{p\left(\mathrm{data}|{M}_0\right)}, $$

where *p*(data | *M*_1_) is the marginal likelihood of *M*_1_. The Bayes factors were computed in R using the Savage-Dickey density ratio representation (Dickey and Lientz 1970; Wagenmakers et al. 2010) and using the *bridgesampling* R package (Gronau et al. 2017a, b). The Bayes factor BF_*i*0_ expresses the evidence in the data for including a particular set of covariates (i.e., the covariates in model *M*_*i*_) against excluding these covariates (i.e., the baseline model *M*_0_). When BF_*i*0_ > 1, the evidence is in favor of including the covariates. When BF_*i*0_ < 1, the evidence is in favor of excluding the covariates. The categories of Jeffreys (1961) are used as benchmarks for the interpretation of the amount of evidence. A Bayes factor greater than 3 or else less than 1/3 represents moderate to substantial evidence, conversely, anything between 1/3 and 3 is only weak or insubstantial evidence. We report the Bayes factors for the different models in comparison to *M*_0_. By transitivity, we may compute other Bayes factor of interest. For instance, the Bayes factor BF_32_, which compares model *M*_3_ with model *M*_2_, may be computed as:

$$ {\mathrm{BF}}_{32}=\frac{{\mathrm{BF}}_{30}}{{\mathrm{BF}}_{20}}=\frac{\frac{p\left(\mathrm{data}|{M}_3\right)}{p\left(\mathrm{data}|{M}_0\right)}}{\frac{p\left(\mathrm{data}|{M}_2\right)}{p\left(\mathrm{data}|{M}_0\right)}}=\frac{p\left(\mathrm{data}|{M}_3\right)}{p\left(\mathrm{data}|{M}_2\right)} $$

Similarly, we find BF_0*i*_ = 1/BF_*i*0_. An additional Bayes Factor was calculated for model *M*_0_ against a null model predicting a mean study effect size of 0 (i.e., *θ* = 0, see below), to quantify the evidence for the effect of condition.

#### Bayesian Model Specification

The models for the cognitive bias and substance use outcomes were based on the hierarchical Bayesian *t*-test approach of Marsman et al. (2017). We used the Bayesian *t*-test model formulation of Rouder et al. (2009), assuming that the observations are normally distributed, and started with expressing the mean in the control condition of a particular study as $\mu -\frac{1}{2}\sigma \delta$ and the mean in the training condition as $\mu +\frac{1}{2}\sigma \delta$, such that the difference in group means is equal to *σδ*. Here *μ* denotes the overall mean of the study outcome, *σ*^2^ the common variance in the two conditions and *δ* denotes a standardized effect size. The idea is to model the effect sizes across studies hierarchically, that is, model them as random effects. We followed Marsman et al. (2017) and assumed that the effect sizes come from a normal distribution with an unknown mean *θ* and variance (i.e., study heterogeneity) *τ*^2^. Instead of including a random effect for participants (i.e., modeling the effect of time of measurement), we used the difference in outcome scores between baseline and follow-up as the dependent variable, with positive values indicating a decrease in the outcome (i.e., a decrease in the strength of the targeted cognitive bias towards substance-related cues relative to control cues, or in substance use), which is in line with the Bayesian paired samples *t*-test approach by Rouder et al. (2009).

Including participant-level and study-level covariates extends the basic model *M*_0_ that we described above. At the study level, this implies that the prior mean *θ* on the effect size of a study *s* is replaced by *θ* + ∑_*i*_*γ*_*i*_*x*_*is*_, where *x*_*is*_ denotes the value for covariate *i* of study *s* and *γ*_*i*_ the associated regression coefficient. Similarly, means at the participant-level are extended by covariates associated to individual outcomes, and the mean for a participant *p* in the control condition of a study *s* is given by

$$ {\mu}_s+{\sigma}_s\left({\sum}_j{\beta}_j{y}_{jps}-{\delta}_s\frac{1}{2}\right), $$

while the mean for a participant *p* in the training condition is

$$ {\mu}_s+{\sigma}_s\left({\sum}_j{\beta}_j{y}_{jps}+{\delta}_s\frac{1}{2}\right). $$

Here *y*_*jps*_ denotes the value for covariate *j* of participant *p* in study *s* and *β*_*j*_ is the associated regression coefficient, where *β*_*j*_ are standardized effects.

The relapse model is a binary outcome analogue for the models of cognitive bias and substance use. The primary difference was that a logistic regression model was used as a starting point with the goal of predicting the chance of relapse. The mean in the control condition of a particular study was then modeled as $\mu -\frac{1}{2}\delta$ and the mean in the training condition as $\mu +\frac{1}{2}\delta$. Note that there was no common variance assumed in the logistic regression model. Apart from that, the relapse model was exactly the same and followed the same steps as the models that were used for the cognitive bias and substance use outcomes.

To complete the Bayesian hierarchical models, we used standard non-informative (Jeffreys’s) priors on the mean *μ*_*s*_ and variance ${\sigma}_s^2$ of a study s (note that ${\sigma}_s^2$was not used in the relapse model), that is, $p\left({\mu}_s,{\sigma}_s^2\right)\propto {\sigma}_s^{-2}$. For the study-level variance *τ*^2^, we used a half-Cauchy prior with scale set to five, whereas for the overall mean *θ*, the participant-level covariates *β*, and the study-level covariates *γ*, we used scaled Cauchy distributions. For the Cauchy priors on the overall mean and the regression coefficients, we have used a scale of 1.0.

Given that Bayesian parameter estimation and testing are sensitive to the specification of the prior distribution, we included a sensitivity analysis for each outcome, estimating all models with a narrower (scale of 0.5) and wider (scale of 2.5) prior on the overall mean and the regression coefficients, hence predicting that relatively large effects are very uncommon and very common, respectively (i.e. is, a prior with scale 2.5 assigns more mass to extreme values than a prior with scale 0.5, which concentrates its mass more on values close to zero). For each outcome, we then plotted the Bayes factors for each model against the baseline model *M*_0_ computed by using the three different priors.

### Supplementary Analyses

The two existing CBM meta-analyses were both carried out within the frequentist statistical framework (Cristea et al. 2016; Mogoaşe et al. 2014). For consistency, we also ran the same hierarchical models within the frequentist framework. The detailed description of the one-stage individual patient data frequentist meta-analysis and of the results is reported in the Supplementary Material.

### Data Availability

Due to the confidentiality and sensitive nature of the collected data, the dataframes including the individual patient data created for the analyses, cannot be shared open access and are available only upon request. The dataframes are solely usable to reproduce the results of the current meta-analysis or to update the meta-analysis to include additional studies published after May 2016. The dataframes will be provided solely under the condition that they cannot be distributed to other parties nor shared open access. For more information about the single studies or to access the individual raw datasets please contact the study authors.

All scripts for both the Bayesian and frequentist analyses are available open access on the Open Science Framework platform at https://osf.io/dbcsz/.

## Results

### Study Selection and Individual Patient Data Obtained

**Fig. 1:**
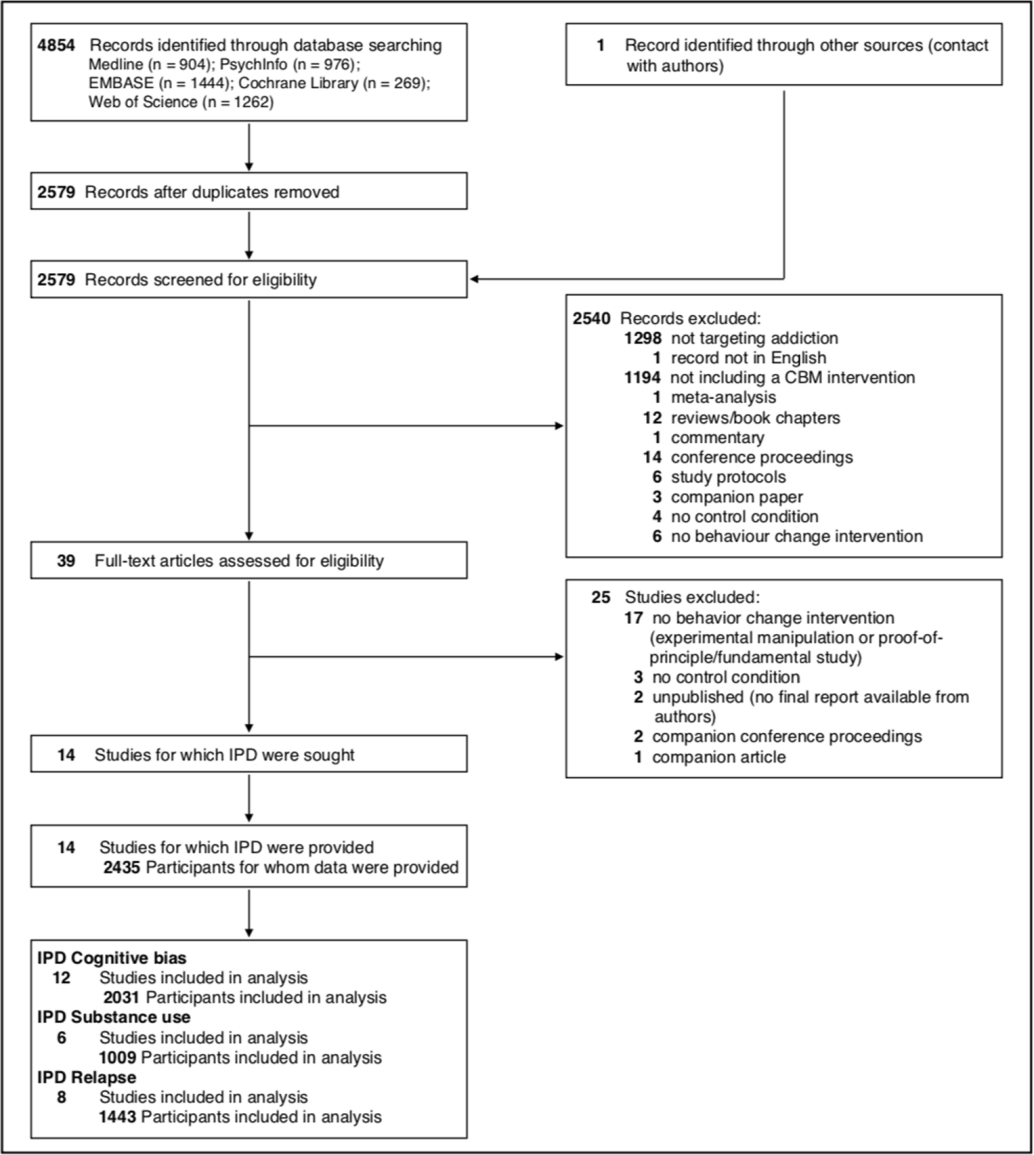
PRISMA-IPD study selection flow

The systematic search resulted in 14 eligible studies out of 2579 search results screened (reference list of included studies reported in Supplementary Material). During the screening process a few conference abstracts could not be matched to a published study. The authors of these conference abstracts were contacted and one additional study was identified through this process. The remaining conference abstracts were associated to previous or later peer-reviewed publications. We obtained individual patient data from all 14 eligible studies, yielding a total of 2435 participants. Figure 1 shows the study selection process.

### Study and Participants Characteristics

Half of the studies targeted alcohol use disorders or problem drinking and the other half tobacco use disorders. Seven studies included an Attentional Bias Modification intervention delivered with an adjusted version of the Visual Probe Task (*n* = 6) or another training paradigm based on the emotional Stroop task, the Alcohol Attention-Control Training Programme (AACTP, *n* = 1; Cox et al. 2015). Six studies deployed an Approach Bias Modification intervention exclusively delivered with the Approach Avoidance Task, and one study included both Attentional Bias Modification and Approach Bias Modification delivered with the AACTP and Approach Avoidance Task training paradigms, respectively (Wiers et al. 2015a, b). No included study targeted evaluative associations or cue-specific response inhibition (Evaluative Conditioning or Selective Inhibition Training), since all these studies were proof-of-principle studies without a behavior change goal (see Allom et al. 2016 and Jones et al. 2016 for syntheses of the results of these proof-of-principle studies).

Most studies involved a parallel-group experimental design, testing the CBM intervention against a control condition, both in combination with TAU (*n* = 8). Two studies tested the combination of Attentional Bias Modification with another training (Clerkin et al. 2016) or a motivational intervention (Cox et al. 2015) in a factorial design. Three of the 14 studies ran online and tested one (Elfeddali et al. 2016; Wittekind et al. 2015) or multiple CBM training varieties (Wiers et al. 2015a, b) as stand-alone interventions. The most used control condition was a sham version of the CBM training (*n* = 9), in the form of a continued assessment using the same task. One study used a placebo training with a different task (Schoenmakers et al. 2010). Three studies included a no-training or wait-list control group (Cox et al. 2015; Eberl et al. 2013; Wittekind et al. 2015), while one study included both types of control condition and found no differential effects on the primary outcomes (Wiers et al. 2011). The majority of studies comprised multiple sessions of CBM (from 3 to 12), except for two delivering one session (McHugh et al. 2010; Wittekind et al. 2015). A description of the main characteristics of each study is presented in Table 1.

Mean age of the 2435 included participants was 42.37 (SD = 12.13, range = 13–80); 1352 (55.5%) were male. All studies included participants selected based on a clinical diagnosis of substance use disorder or on patterns of substance consumption indicative of abuse. The mean severity of substance use problems was 4.62 (SD = 2.38, range = 0–10) on the FTND for tobacco studies and 23.18 (SD = 8.05, range = 0–40) on the AUDIT, 43.15 (SD = 27.24, range = 5–128; Clerkin et al. 2016) on the Drinker Inventory of Consequences, and 12.49 (SD = 8.44, range = 0–42; Cox et al. 2015) on the Short Index of Problems, for alcohol studies. The amount of training sessions across studies ranged from 1 to 12, while the amount of training trials per session from 100 to 573. When data were available, participants completed on average a total of 1006.72 (SD = 827.08) training trials, independently from the training condition they were assigned to, which is equivalent to a mean of 5.03 sessions including 200 trials per session.

### Risk of Bias Assessment

**Fig. 2:**
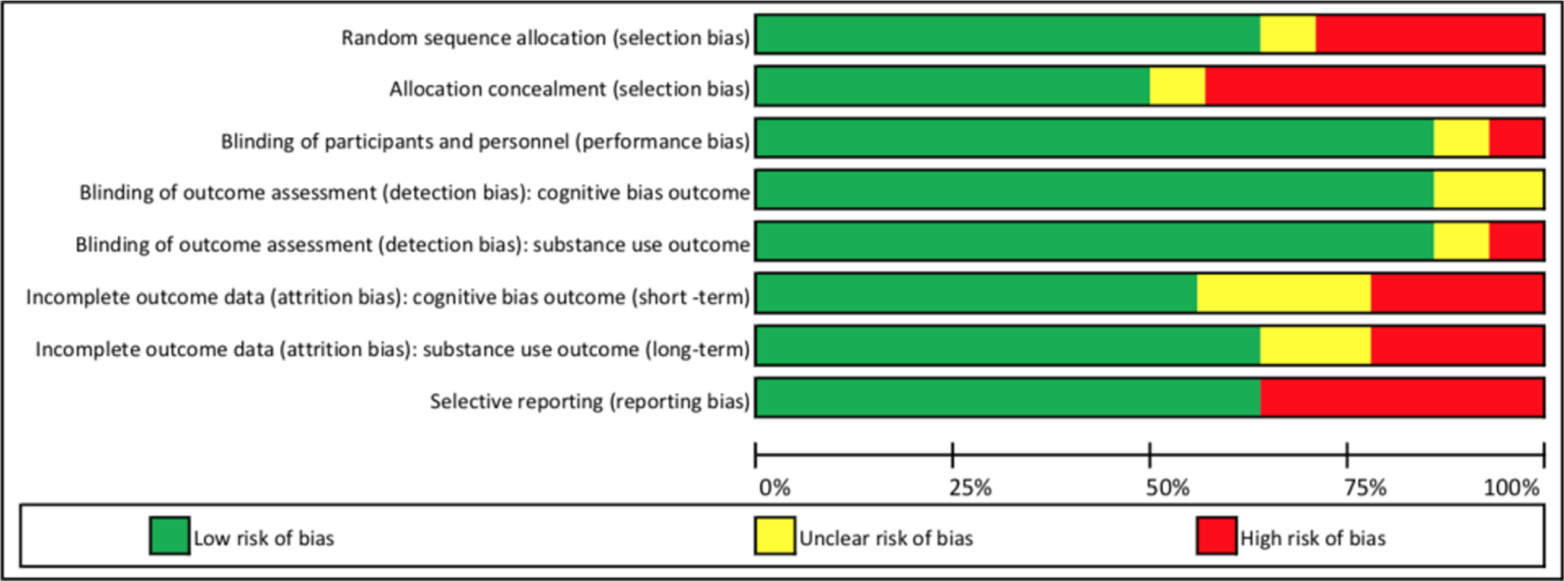
Summary of the risk of bias evaluations for the 14 included studies. Note that the evaluation of attrition bias for the substance use outcome includes both reduction in substance use and relapse rate outcomes

Study quality varied over the items of the Cochrane risk of bias tool but there was generally a low risk of bias (see Fig. 2 for the risk of bias summary graph and Table S10 in the Supplementary Materials for a detailed overview of the information supporting risk of bias judgments for each criterion across all studies). A study was evaluated as having an unclear risk of bias for one or more items when the information provided in the paper or by the authors was not sufficient to make a judgment. For several studies (*n* = 9) the assignment of participants to the condition was random or randomly stratified. Four studies used an assignment strategy that did not involve a random component, while for one study the provided information was not sufficient to make a judgment. Only half of the studies (*n* = 7) implemented a successful concealment of the randomization sequence, while in six this was not done or was not possible. Though all participants were aware that the goal of the intervention was behavior change, blinding of participants and study personnel to the allocation of condition was implemented in most studies (*n* = 11). The risk for assessor (detection) bias in both cognitive bias and behavioral outcome(s) was generally low (*n* = 12 for both outcomes). Cognitive bias(es) were assessed with reaction-time computerized tasks, and substance use with self-report measures. Following the Cochrane guidelines, studies not addressing an outcome included in the meta-analysis were evaluated as having an unclear risk of performance bias for that outcome (*n* = 2 for cognitive bias and n = 1 for substance use outcomes). Eight and nine studies used some form of imputation or coding criteria to handle missing data in the cognitive bias and behavioral outcomes, respectively, or differences in attrition between conditions were non-significant, indicating a low risk of attrition bias for both types of outcomes in only 57 and 64% of the studies. For each outcome, three studies included completers only or applied stringent exclusion criteria without running sensitivity analyses, resulting in a high risk of bias. Finally, nine studies were evaluated as having a low risk of reporting bias due to either the presence of some form of pre-registration of the study outcomes (e.g., protocol article or registration in official registry of randomized clinical trials) or through formal confirmation from the authors. Five studies did not include one or more outcomes in the final report and have been evaluated at high risk for reporting bias.

### One-Stage Bayesian Individual Patient Data Meta-Analysis

#### Change in Cognitive Bias

**Fig. 3:**
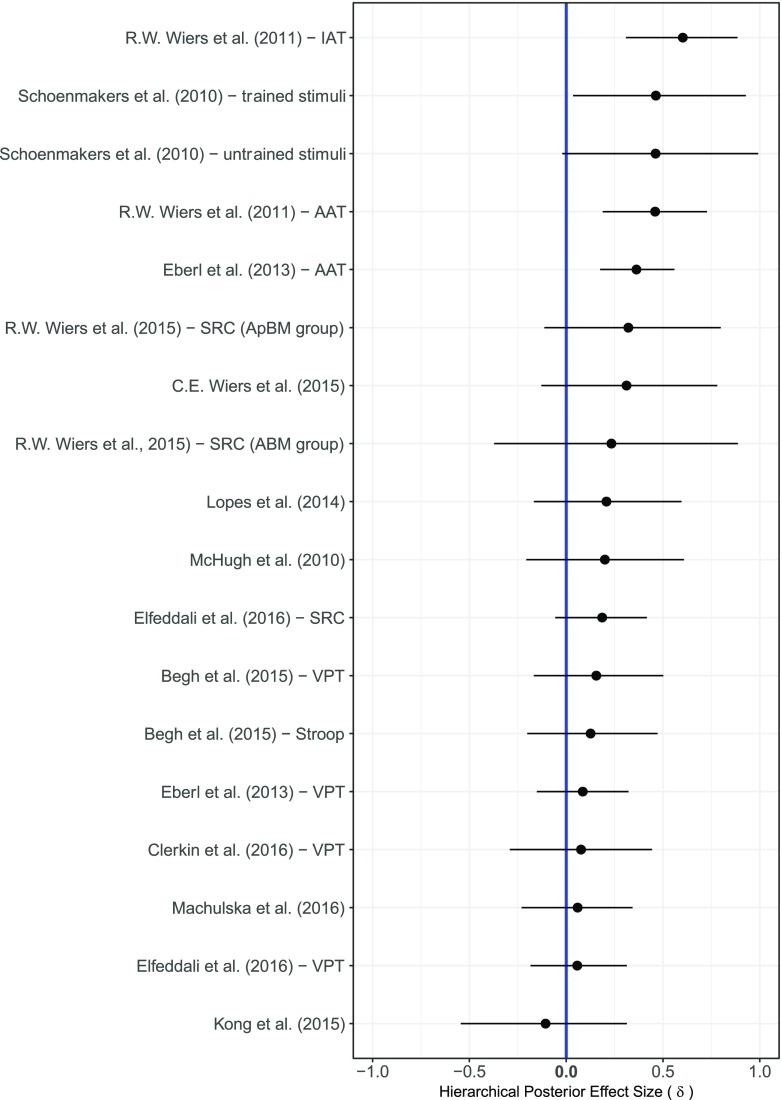
Results of the Bayesian parameter estimation of the effect sizes *δ* for each of the 18 comparisons included in the analysis of the cognitive bias outcome. The effects sizes were estimated using the baseline model *M*_0_. The posterior means are indicated as dots and the 95% central credible intervals as horizontal lines

Figure 3 shows the forest plot of the effect sizes *δ* separately for each of the 18 comparisons in the baseline model *M*_0_ of the cognitive bias outcome. The estimated effect sizes were generally small, and many comparisons yielded credible intervals that were relatively wide—an indication that there remains considerable uncertainty about the true value of the effect size. With small average effects and wide credible intervals, only four out of the 18 comparisons 95% central credible intervals did not overlap with zero. The overall effect size *θ* for the baseline model *M*_0_ was small, the posterior mean was equal to 0.23 with a 95% credible interval ranging from 0.06 to 0.41. Similarly, the between comparison heterogeneity *τ*^2^ was also small with a posterior mean equal to 0.09 and a 95% credible interval ranging from 0.02 to 0.25. The Bayes factor of the null model without including the effect of condition against model *M*_0_ was equal to 0.47, showing that there is no substantial evidence in favor or against either model.

Table S1 in the Supplementary Materials includes the parameter estimation results for models *M*_0_ to *M*_6_ in the cognitive bias outcome analysis. The overall effect size *θ* remained small for each of the six models. Furthermore, there was a small negative effect for type of addiction of about −0.20 in models *M*_3_ to *M*_6_, which implied that the effect sizes in alcohol CBM studies were slightly larger, on average, than tobacco studies. Note that the effect of type of addiction was roughly the same as the overall effect size, which implies that the expected effect in tobacco studies is about zero and the expected effect for alcohol related comparisons is about 2*θ* ≈ 0.4. All other effects were small, negative and uncertain, with posterior means ranging from −0.11 to −0.03 and 95% credible intervals overlapping with zero.

Table S1 also shows the log Bayes factor for each of the six models, comparing their predictive accuracy against model *M*_0_. All log Bayes factors were negative (i.e., Bayes factor < 1), expressing evidence in favor of model *M*_0_. There was a substantial amount of evidence against models *M*_3_, *M*_4_, *M*_5_ and *M*_6_, and in favor of the baseline model *M*_0_. For example, it is about e^4.32^ ≈ 75 times more likely that the data came from model *M*_0_ instead of model *M*_3_. Furthermore, the Bayes factors for models *M*_1_ and *M*_2_ are relatively close to zero, which implies that there is little evidence either in favor or against them in comparison to the basic model *M*_0_ (i.e., the log Bayes factors are between log(1/10) = −2.30 and log(10) = 2.30).

As the aim was to compare different models, which can be done through transitivity of Bayes factors when computed on the same data, participants with missing values on the covariates had to be excluded from the analyses. Since there were no data available on the severity of substance use covariate for two comparisons (Schoenmakers et al. 2010), we would have had to exclude these comparisons from all analyses in order to compare across models *M*_1_ to *M*_7_. Instead of excluding the data from these comparisons across all models, we opted to include all available data across models *M*_1_ to *M*_6_ including all comparisons, and estimate model *M*_7_ separately. The 18 comparisons included 3369 observations for the analysis of cognitive bias data, with 2112 observations without any missing values for the covariates that were used in the models reported in Table S1. An additional 92 observations were omitted for the analysis of model *M*_7_, of which 69 came from the two excluded comparisons.

We report the results for model *M*_7_ on the reduced dataset in Table S2 (Supplementary Materials). When comparing the estimated effects of *M*_7_ with the estimated effects of *M*_6_ reported in Table S1, we found substantial differences. For instance, the overall effect size and the effect of type of addiction roughly halved their values, and the 95% central credible intervals for each effect overlapped with zero in *M*_7_. Severity of substance use problems showed almost no effect, with a posterior mean equal to −0.01 and a 95% credible interval ranging from −0.06 to 0.03. Further, the log Bayes factor strongly supported the baseline model *M*_0_ (both marginal likelihoods were computed on the reduced dataset). To summarize, CBM was found to modestly reduce cognitive bias, although this effect was associated with much uncertainty, and was not affected by moderators, with the exception that reduction in cognitive bias after the training intervention is more likely to be observed for bias toward alcohol, but not toward tobacco.

**Fig. 4:**
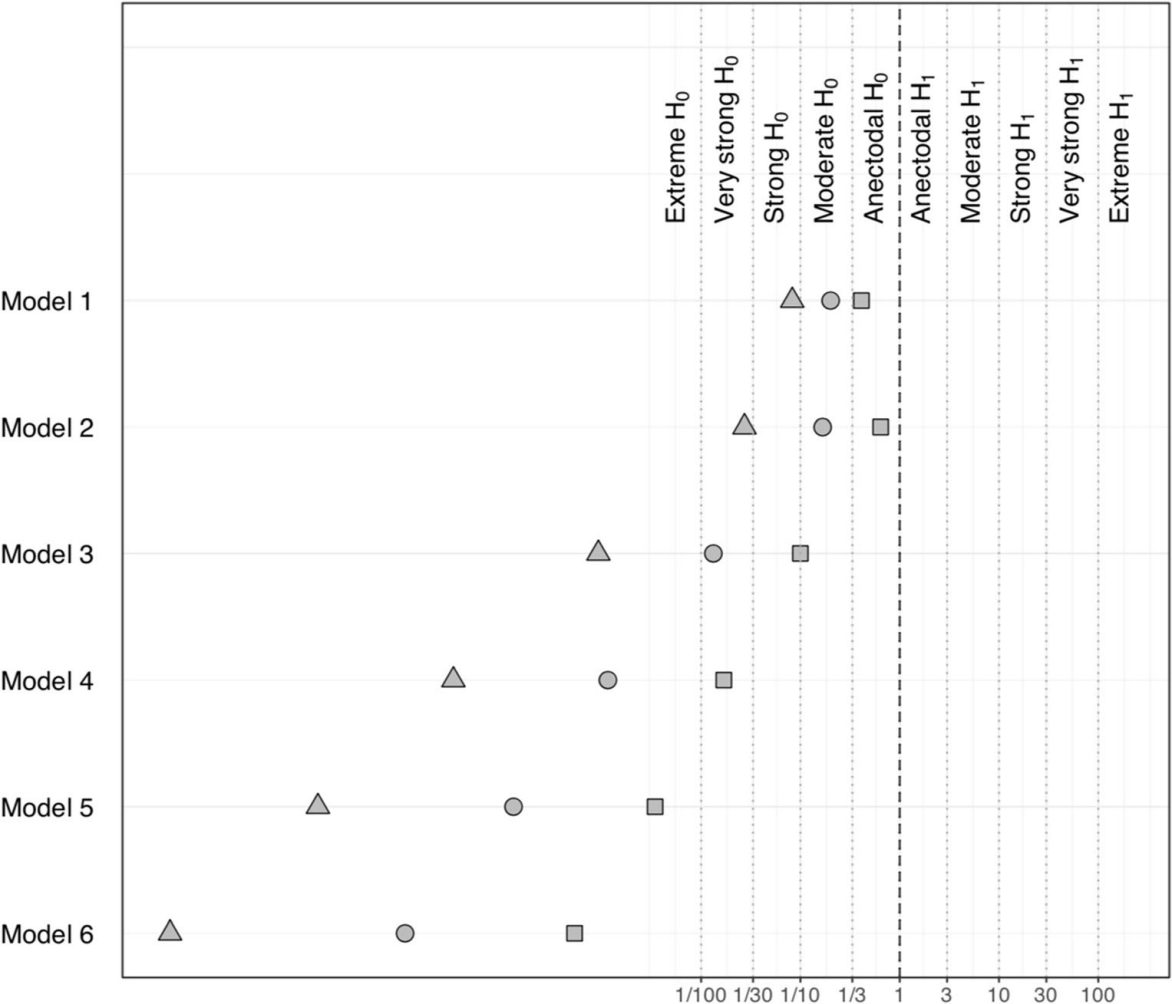
Sensitivity analysis of cognitive bias outcome showing Bayes factors for models *M*_1_ – *M*_6_ against model *M*_0_ computed with the three different prior distributions on the main parameters of interest: primary prior distribution (scale of 1; circle), wider prior (scale 2.5; triangle) and narrower prior (scale 0.5; square). The direction of the hypothesis refers to the two-sided BF10. The top margin indicates the evidence categories proposed by Jeffreys (1961)

Figure 4 displays the results of the sensitivity analyses carried out with the two additional prior distributions. The Bayes Factor for models *M*_1_ – *M*_6_ (against model *M*_0_) further corroborate the lack of evidence for the inclusion of any of the covariates and moderators in models *M*_1_ to *M*_6_, relative to the simpler model *M*_0_, since they all range in the region of acceptance of H_0_ (i.e., model *M*_0_ is more plausible**).** The pattern of results is not different when using a narrower or wider prior, as shown by the monotonic relationship of Bayes factors values across all models. Due to the different number of observations included in model *M*_7_, the same sensitivity analysis was conducted separately, with a similar trend in the results.

#### Reduction of Substance Use

**Fig. 5:**
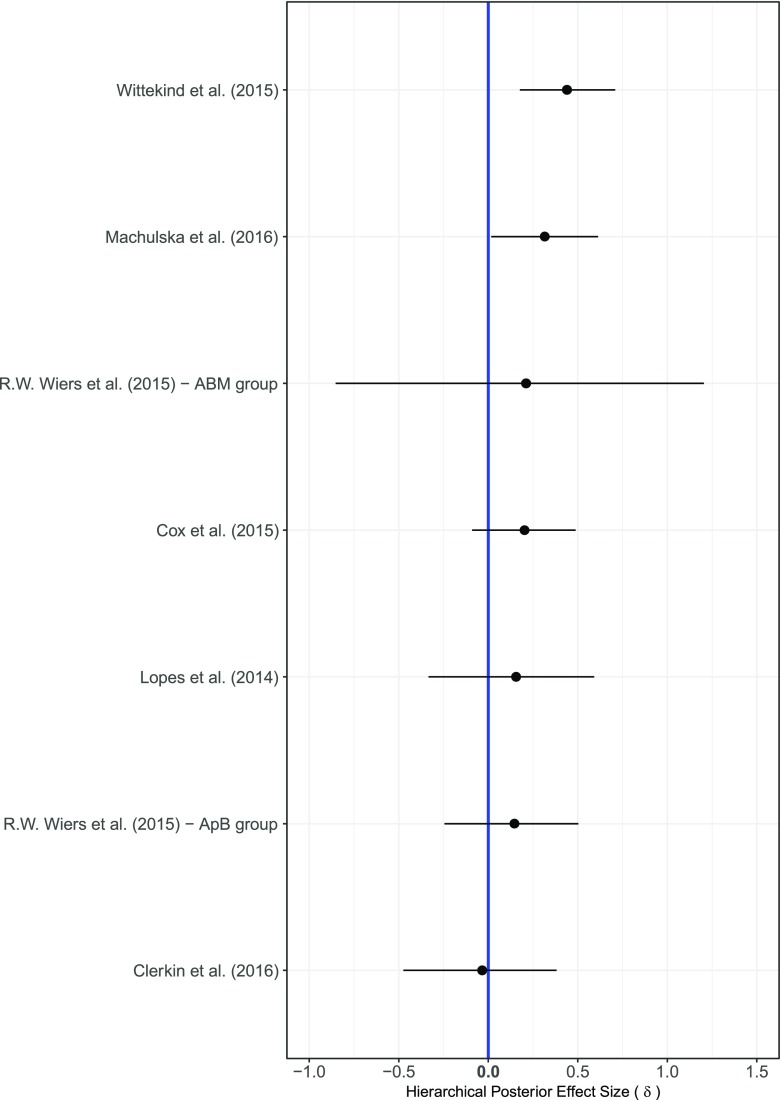
Results of the Bayesian parameter estimation of the effect sizes *δ* for each of the seven comparisons included in the analysis of the substance use outcome. The effects sizes were estimated using the baseline model *M*_0_. The posterior means are indicated as dots and the 95% central credible intervals as horizontal lines

Figure 5 shows the forest plot for the effect sizes *δ* separately for each of the seven comparisons in the baseline model *M*_0_ for the reduction in substance use outcome. With the exception of the study by Wittekind et al. (2015) the estimated effect sizes were all small, with many studies yielding relatively wide credible intervals. Two out of the seven 95% central credible intervals did not overlap with zero. The overall effect size *θ* in the baseline model *M*_0_ was small, the posterior mean was equal to 0.19 with a 95% central credible interval ranging from −0.23 to 0.58. The between study heterogeneity *τ*^2^ was also found to be small with a posterior mean equal to 0.22 and a 95% credible interval ranging from 0.01 to 1.20. The Bayes factor for the null model not including the effect of condition against model *M*_0_ was equal to 3.06, showing that there is moderate evidence against *M*_0_.

The parameter estimation results for models *M*_1_ to *M*_6_ in the reduction of substance use analysis are reported in detail in Table S3 in the Supplementary Materials. In each of the six models the overall effect size *θ* remained small. Moreover, even though several small effects were estimated, e.g., amount of completed training trials, the 95% central credible interval for each effect was relatively wide and overlapped with zero. The log Bayes factors that are reported in Table S3 are in line with these results. Except for the Bayes factor contrasting models *M*_1_ and *M*_0_, which reveals little evidence in favor or against model *M*_1_ when compared to *M*_0_, all Bayes factors show strong support for the baseline model *M*_0_.

Similar to the cognitive bias outcome, we analyzed *M*_7_ separately (see Table S4 in the Supplementary Materials). The seven comparisons for the substance use outcome included 1064 observations, with 768 observations having no missing values for the covariates used in the models reported in Table S3**.** An additional eight observations were omitted for the analysis of model *M*_7_ due to missing data on the severity of substance use covariate.

The estimated effects of *M*_7_ were similar to the estimated effects of *M*_6_ reported in Table S3. We found a small main effect of severity of substance use (its posterior mean was equal to 0.18 with a 95% central credible interval ranging from 0.11 to 0.26), indicating a positive relationship between the increase in severity of substance use problems and increase in consumption at follow-up. The reported log Bayes factor also suggested that there was substantial evidence to include the effect of severity of substance use in *M*_7_ relative to model *M*_6_. The relative predictive adequacy of the most complex model including the effect (model *M*_7_), compared to the simplest model excluding the effect (model *M*_6_), was computed as follows

$$ {\mathrm{BF}}_{76}=\frac{{\mathrm{BF}}_{70}}{{\mathrm{BF}}_{60}}=\frac{\frac{p\left(\mathrm{data}\ |\ {M}_7\right)}{p\left(\mathrm{data}\ |\ {M}_0\right)}}{\frac{p\left(\mathrm{data}\ |\ {M}_6\right)}{p\left(\mathrm{data}\ |\ {M}_0\right)}}=\frac{p\left(\mathrm{data}\ |\ {M}_7\right)}{p\left(\mathrm{data}\ |\ {M}_6\right)}. $$

Based on the log Bayes factor values reported in Tables S3 and S4, logBF_76_ = *e*^−0.11 + 8.19^ ≈ 3,229.23, which indicated overwhelming evidence in favor of including the covariate effect of severity of substance use problems. Moreover, this comparison assumed that the removal of the cases with missing data on the severity of substance use covariate could be safely ignored. Another way of expressing the evidence is to argue that there was substantial evidence against including any of the effects in model *M*_**6**_ in Table S3, while this is certainly not the case for the effects in model *M*_7_ reported in Table S4. In short, although a main effect of severity of substance use was identified, the Bayesian analysis of the substance use outcome showed no reliable evidence in support for a differential effect of training condition over the decline in substance use between baseline and follow-up.

**Fig. 6:**
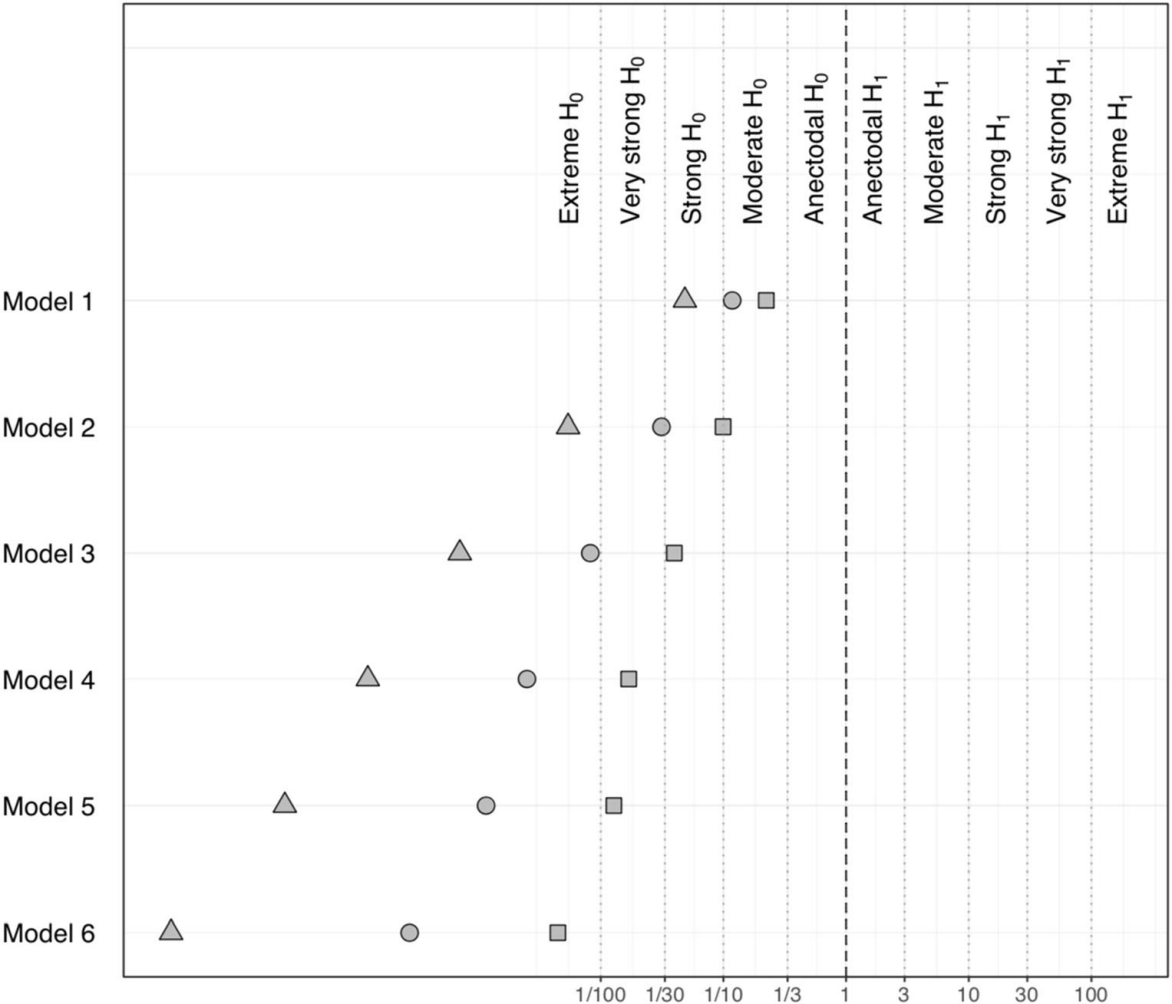
Sensitivity analysis of reduction in substance use outcome showing Bayes factors for models *M*_1_ – *M*_6_ against model *M*_0_ computed with the three different prior distributions on the main parameters of interest: primary prior distribution (scale of 1; circle), wider prior (scale 2.5; triangle) and narrower prior (scale 0.5; square). The direction of the hypothesis refers to the two-sided BF10. The top margin indicates the evidence categories proposed by Jeffreys (1961)

Figure 6 displays the results of the sensitivity analyses carried out with the two additional prior distributions. The Bayes factor for models *M*_1_ – *M*_**6**_ (against model *M*_**0**_) further corroborate the lack of evidence for the more complex models since all Bayes factors indicate evidence in favor of H_0_ (i.e., the data are more plausible under *M*_**0**_**).** The pattern of results is not different when using a narrower or wider prior, as shown by the monotonic relationship of Bayes factor values across all models. The same sensitivity analysis for model *M*_7_ showed a similar trend.

#### Relapse Rate

**Fig. 7:**
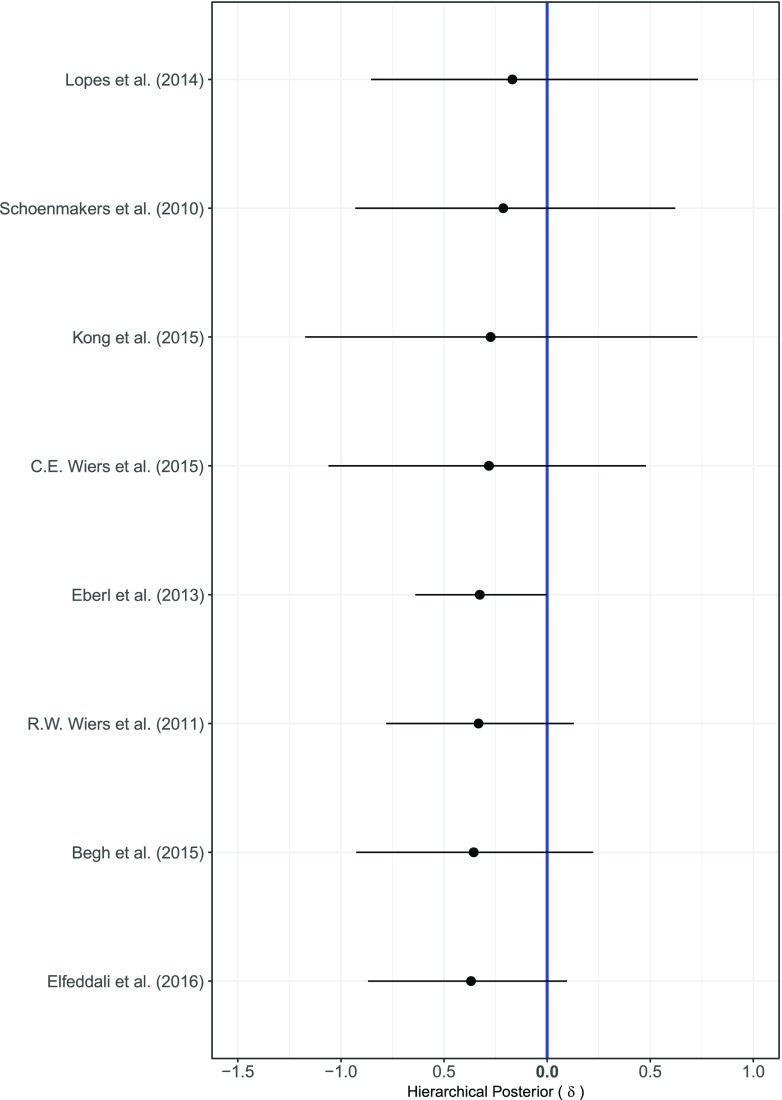
Results of the Bayesian parameter estimation of the mean log odds difference *δ* between training and control condition for each of the eight comparisons included in the analysis of the relapse outcome. The intercepts were estimated using the baseline model *M*_0_. The posterior means are indicated as dots and the 95% central credible intervals as horizontal lines

Figure 7 shows the forest plot of the log odds difference *δ* between the training and the control condition for each of the eight comparisons for the relapse outcome. All estimated effects were negative, indicating that there was a positive effect of training (i.e., a lower probability of relapse), yet all of the 95% central credible intervals overlapped with zero. The overall effect *θ* was also small and negative, the posterior mean was equal to −0.27 with a 95% credible interval ranging from −0.68 to 0.22. The between study heterogeneity *τ*^2^ was also small with a posterior mean equal to 0.21 and a 95% credible interval ranging from 0.01 to 1.15. The Bayes factor of for the null model not including the effect of condition against model *M*_0_ was equal to 2.06, showing that there is no substantial evidence in favor of either model.

The parameter estimation results for models *M*_0_ to *M*_6_ revealed a main positive effect of number of training trials and a negative interaction effect with training condition on the probability of relapse in model *M*_6_ (estimates are reported in detail in Table S5 in the Supplementary Materials). The posterior mean of the log odds for the main effect of number of training trials was equal to −1.29 with a 95% central credible interval ranging from −1.89 to −0.66, while the posterior mean of its interaction with condition was equal to 0.88 with a 95% credible interval ranging from 0.27 to 1.54. Even though there is still much uncertainty in both effects, it appears that completing more training trials leads to 72% lower probability of relapse. However, when considering the interaction effect with the training condition, this effect seemed to be attenuated in the training condition, which showed [(*e*^( − 1.29 + (.87 × 0.5)^) − 1] ∗ 100 ≈ 57% lower chance of relapse as the amount of completed training trials increases, while the control condition showed [(*e*^( − 1.29 + (.87 ×  − 0.5)^) − 1] ∗ 100 ≈ 82% lower chance to relapse.

All other effects, except for number of training trials in *M*_5_ and its interaction with condition in *M*_6_, showed 95% credible intervals overlapping with zero. Interestingly, there is evidence in favor of including both the effect of the amount of completed training trials, as for its interaction with condition. Based on the log Bayes factor values in Table S5 we found BF_54_ = *e*^−1.36 + 5.14^ ≈ 43.81 and BF_65_ = *e*^1.47 + 1.36^ ≈ 16.95, both indicating support for including the effect.

Also for relapse rate, we analyzed model *M*_7_ separately from models *M*_1_ to *M*_6_. The eight comparisons for the relapse outcome included 1424 observations, of which 1411 with no missing values for the covariates were used in the models *M*_1_ to *M*_6_ reported in Table S5. An additional 54 observations were omitted for the analysis of model *M*_7_.

Table S6 reports the results for model *M*_7_ after excluding these observations**.** The estimated effects of *M*_7_ were very similar to the estimated effects of *M*_6_. The effect of severity of substance use was very small with a posterior mean equal to 0.13 and a 95% central credible interval ranging from −0.01 to 0.26. Since the Bayes factors in Table S5 and Table S6 have been computed on different datasets the comparison of their values should be done with caution. The Bayes factor in Table S6 suggests that there is no evidence in favor or against *M*_7_ when compared to the baseline model *M*_0_. Further, the amount of evidence is lower than the amount of support that *M*_6_ received. This result suggests that there is no evidence for the inclusion of addiction severity. In sum, the Bayesian analysis showed a small albeit unreliable effect of CBM on relapse and a positive moderation effect of the amount of trials completed by participants. However, this moderation effect was stronger in the control condition, with a greater reduction in relapse rate for the control relative to the training condition. Yet, there is no substantial evidence against or in favor of these effects.

**Fig. 8:**
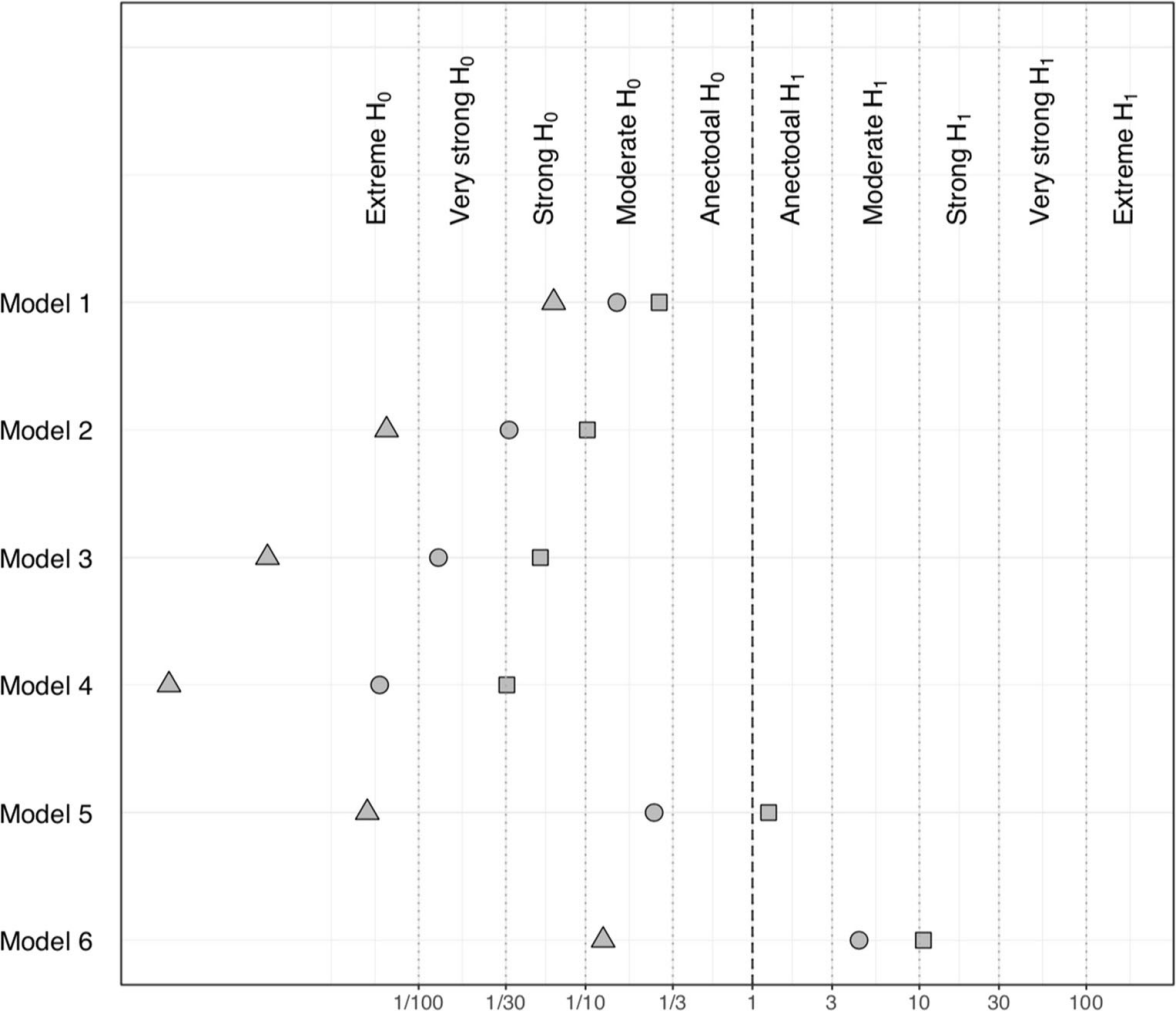
Sensitivity analysis of relapse outcome showing Bayes factors for models *M*_1_ – *M*_6_ against model *M*_0_ computed with the three different prior distributions on the main parameters of interest: primary prior distribution (scale of 1; circle), wider prior (scale 2.5; triangle) and narrower prior (scale 0.5; square). The direction of the hypothesis refers to the two-sided BF10. The top margin indicates the evidence categories proposed by Jeffreys (1961)

Figure 8 displays the results of the sensitivity analyses carried out with the two additional prior distributions. The Bayes factor for models *M*_1_ – *M*_**5**_ (against model *M*_**0**_) corroborate the lack of evidence for such models since all Bayes factors range in favor of H_0_ (i.e., data are more plausible under *M*_**0**_**).** However, in line with the parameter estimate results, the inclusion of the moderation effect of amount of completed training trials shifted the evidence in favor of model *M*_6_**.** The pattern of results is not different when using a narrower or wider prior, except for a spreading effect towards more extreme values (as implied by using a narrower and wider prior distribution for the mean effect and regression coefficients). The same sensitivity analysis for model *M*_7_ showed a similar trend of results as for model *M*_6_.

### Supplementary Analyses

The one-stage frequentist individual patient data meta-analysis included the estimation of models *M*_0_ to *M*_6_ on the same dataset for the three outcomes. A detailed description of the data analysis and of the results is reported in the Supplementary Materials. The analysis of the cognitive bias outcome evidenced a small effect of training condition on the change in cognitive bias from baseline to post-intervention (*β* range = 0.10–0.14, *p’*s ≤ .01; Table S7). This effect was not affected by any of the study- or participant-level covariates or moderators. No significant effect of condition on the difference in substance use between baseline and follow-up was found (Table S8). For relapse rate, the results appeared to be more complex (Table S9). The most complex model *M*_7_ showed the best fit to the data, including a main effect of type of addiction (higher chance of relapse for tobacco use disorder) and type of CBM training (lower chance of relapse when deploying Approach Bias Modification training) on relapse rate, but no significant effect of condition on relapse rate. Note that in this model two comparisons were not included due to missing data. However, similar results were observed in the next best fitting and more parsimonious model *M*_5_, including all study comparisons. Training condition significantly affected relapse rate only in those models that did not include the number of completed training trials as a covariate (i.e., models *M*_0_, *M*_1_, *M*_2_, *M*_3_ and *M*_4_), all indicating around 16% lower chance to relapse in the training group (ORs ≈ 0.84, *p*s < .05). However, these models showed a very poor fit to data.

## Discussion

In this study, we examined the effectiveness of CBM interventions for addictive behaviors, specifically for the treatment of alcohol and tobacco use problems, by conducting a meta-analysis of studies explicitly testing CBM as a behavior change intervention with the targeted recipient population (i.e., individuals with a clinical diagnosis of substance use disorder or suffering from substance use problems who were aware that the goal of the intervention was behavior change). The goal of the meta-analysis was twofold. First, we aimed at testing whether CBM interventions have a global impact on both the targeted cognitive bias(es) and on substance use behavior, in terms of reduction in drug consumption and relapse rate. Second, given the variety of CBM paradigms, training program characteristics and dosages, and differences across people meeting criteria for alcohol use disorders, typically patients with comorbid problems, and tobacco use disorders, typically well-functioning adults who have a problem quitting smoking, we aimed at evaluating the impact of relevant covariates and moderators of CBM interventions effects. To this aim, we conducted an individual patient data meta-analysis to test a series of hierarchical models progressively including multiple study- and participant-level moderators of CBM effect sizes. Additionally, we conducted the meta-analysis in the Bayesian statistical framework, in order to explicitly quantify and test the available evidence in favor or against CBM.

In the 14 studies meeting the eligibility criteria, CBM interventions were found to have a small, albeit unreliable, overall effect on cognitive bias directly after the completion of the training intervention. When the goal was reducing substance use, no differential effect on substance use was observed, whereas when the outcome was abstinence, an overall small, albeit very uncertain, effect was observed in the medium-to-long term, as demonstrated by the extremely wide 95% credible intervals of the effect sizes in the Bayesian results.

When examining the effect of the covariates and related interaction effects with training condition, very weak evidence was found regarding the inclusion of covariates and moderators in the models, as shown by the majority of Bayes factors favoring the simplest models excluding all moderators. None of the moderators appeared to have a substantial impact on the CBM effects on the outcomes, with the exception of amount of completed training trials in the relapse analyses. The latter moderator was added to account for the individual variability in training adherence, but also to account for the inter-study variability in the amount and length of training sessions of the included studies, as shown in Table 1. The inclusion of such moderator was also in line with the results of a post-hoc follow-up study of Eberl et al. (2014), which re-analyzed a sub-set of participants in Eberl et al. (2013) and showed large individual differences in learning effects along multiple sessions, emphasizing the importance of including at least five sessions of training when delivering an Approach Bias Modification intervention.

Indeed, the amount of completed training trials appeared to both improve the likelihood of the data under the specified model *M*_6_, outperforming the baseline model *M*_0_, *and* to moderate the effect of CBM on relapse rate. Specifically, although completing more training trials appeared to reduce the chance of relapse, when examining the effect of the interaction with training condition, it appeared that it might actually result in larger effects in the control condition, thus reducing, by comparison, the beneficial effects of the real training (i.e., smaller decrease in the chance of relapse in the training *compared to* the control condition). Note that in the same analyses carried out with the frequentist approach (see Supplementary Material), the amount of completed training trials variable appeared to be irrelevant despite increasing the goodness of fit of the models including it, since it did not substantially affect the training condition effects on the relapse outcome (OR’s = 1.00). As a matter of fact, in the frequentist analysis of relapse rate, the effect of training condition disappeared after the inclusion of the main and interaction effects of amount of training trials in models *M*_5_ to *M*_7_, whereas it was still significant in the simpler models *M*_0_ to *M*_4_.

In ten out of the 14 studies, participants assigned to the control condition completed a sham training, which exposed them to the same substance-related and neutral stimuli presented in the training condition, though with no stimulus-response contingency (e.g., a continuous assessment task with substance-related and neutral stimuli pulled and pushed equally often). The results seem to suggest that the continuous exposure to the same substance-related stimuli presented without any task contingency may induce a greater decrease in the chance of relapse for participants in the control condition, compared to those completing the active CBM training. Due to the absence of any relation between stimulus category and the actual task response, that is, stimuli are equally pushed and pulled in Approach Bias Modification or equally replaced by a probe in Attentional Bias Modification trainings, it may be possible that participants in the control condition learn to ignore the contents of the stimuli presented and simply focus on performance, which in turn may translate into a lower reactivity toward triggers of substance use behavior. This hypothesis suggests that there might be two mechanisms at work in CBM, firstly, an active re-training mechanism, where the dominant cue-induced response-tendency is changed to another dominant response-tendency, which appears to happen quickly--as shown by the small CBM effect on changes in cognitive bias at conclusion of the intervention--, and a more general extinction-like process making patients less sensitive to the motivational meaning of the addiction-relevant cues (cf. den Uyl et al. 2017). Testing this hypothesis would require a study design focussing on both the clinical effectiveness and working mechanisms of CBM, by comparing a CBM intervention against both an (active) sham training control and a full control condition with no training (e.g., treatment as usual), and by evaluating potential mediation effects of changes in cue reactivity towards substance-related cues across the three conditions. Note that one of the largest studies included in the meta-analysis originally contrasted an Approach Bias Modification training against two such control conditions, with no differential effect on the outcomes, although no assessment of cue reactivity was included (Wiers et al. 2011); hence, the control conditions were merged in the current meta-analysis.

Although the majority of included studies used a sham training as comparator, one of the four remaining studies used a different placebo task unrelated to the training paradigm, while three included a no-training or wait-list condition, with participants completing zero training trials. The large amount of zeros observed in the amount of completed training trials variable for the latter three studies, may have biased its moderation effects of including condition, due to a possible underlying confounding effect of different control conditions. Further, some studies had some missing data points in this moderator--,which led to the exclusion of 133 observations for the model including it, and in some studies all participants completed the same amount of training sessions, decreasing the degree of variability in the data. Due to the small number of studies, it was not possible to add type of control condition as an additional study-level moderator, which could have clarified the difference in the comparator condition and shed light on the hypothesis of different working mechanisms at play in the sham training condition. Further, we could not run a sensitivity analysis excluding the studies using a different control condition due to the additional loss in cases, which would further reduce the amount of available evidence for the main intervention effect.

Although interesting from a theoretical and experimental point of view, the considerable uncertainty associated with these results prevents definite conclusions. Nonetheless, the choice of the optimal comparator in effectiveness studies plays a crucial role in estimating the true effects of an intervention (Blackwell et al. 2017; Hertel and Mathews 2011). A sham version of the CBM training would at first sight seem to be the ideal comparator to evaluate the relative efficacy of CBM, as it allows the researcher to isolate the underlying alleged training mechanism, while keeping the procedural features and exposed contents of the training constant (i.e., minimal credible intervention). However, such a control condition may be sensitive to effects of general exposure mechanisms and extinction-like or desensitization learning processes, as mentioned above. Moreover, there may be placebo- or nocebo-effects at play (note that for Attentional Bias Modification, participants in both conditions typically believe they are in the control condition, and experience the training as rather meaningless, Beard et al. 2011). This is an issue for clinical applications of CBM programs. One approach could be to explain the idea behind CBM, but there is some evidence from anxiety treatment studies that this might be counterproductive (Grafton et al. 2014), although in the addiction field no differences were found between a more and less explicit experimental condition (Wiers et al. 2011). One issue with this approach relates to the blinding of conditions as is preferred in clinical research (Boutron et al. 2008; Schulz and Grimes 2002; Wiers et al. 2018). In addition, the contents of CBM could be better aligned to the contents of the accompanying therapy, typically cognitive behavioral therapy, by personalizing not only the addiction-relevant cues, but also the alternatives (see Kopetz et al. 2017, for a proof-of-principle study). However, this approach will further complicate blinding.

An additional concern is related to the fact that even in a between-subject design, the contents of the control condition (i.e., sham training) may not be sufficiently contrasted with that of the training condition, which may induce a certain lack of differentiation (i.e., dependence) between these two conditions. For example, if the procedural features between the active training and the sham training are too similar to each other, in addition to not detecting a differential effect between the two, we may consider that they are so similar that scores on one condition may predict scores on the other condition, or that the chance of detecting an already small training effect is further shrunk by a “diluted” training effect in the sham condition, due to the exposure of addiction-relevant stimuli in the trained-response format in half of the trials (e.g., avoid response in Approach Bias Modification or shift attention away in Attentional Bias Modification; cf. Salemink et al. 2014). The latter situation falls within the broader discussion into the selection of the most appropriate control comparator based on the research question of interest, which highlights the inefficiency and poor utility of sham training as a truly neutral or placebo comparator when addressing the clinical utility of CBM (Blackwell et al. 2017; Kakoschke et al. 2018), due to the potential, albeit “diluted” active effects mentioned above. Testing specific training mechanisms underlying therapeutic effects, evaluating the efficacy of CBM as an adjunct intervention added to an existing treatment, or as a first line low-threshold intervention program, are different research questions implying different choices in terms of what the appropriate control condition should include (for an extensive discussion see, Blackwell et al. 2017).

Despite the small effects on cognitive bias and relapse in both statistical frameworks, these effect sizes were found to be extremely unreliable and uncertain. Indeed, the amount of CBM studies qualifying as “true” behavior change studies is still very small (*n* = 14), with consequent limited and inconsistent empirical evidence in favor or against CBM, as confirmed by the values of Bayes factors for the simpler model including the effect of training condition. During the selection process we excluded 23 additional CBM studies because they were not set up to evaluate the therapeutic effects of CBM as a behavior change intervention. In many of the screened CBM reports the presentation of the study was ambiguous and it had been necessary to contact the authors asking clarifications about the original goal of the study: whether the primary focus of the respective study was aimed at testing mechanisms of bias-change (proof-of-principle studies in students), or whether it was behavior change (often in patients, but in some cases also in students). Hence, for future studies, it would seem imperative to clearly define the primary goals as clinical or experimental (cf., Wiers et al. 2018).

A last remark addresses an often-overlooked CBM training parameter, that is, the interval between training sessions. Learning and consolidation effects are not only dependent on on-line learning, that is, within-session active learning based on repetitive practice, but also on off-line learning processes, namely, between-session passive learning based on consolidation processes. Therefore, the time interval between training sessions is very likely to play a role in the consolidation of the training effects in the long-term memory (e.g., Abend et al. 2014). Unfortunately, the schedule of training sessions and the evaluation of participant adherence to training schedules has not been systematically addressed in both the design of CBM training protocols and the evaluation of their effects, which limits the exploration of the effects of this additional study-level parameter into an aggregate analysis of CBM effects.

In contrast to the substantial number of proof-of-principle studies, it is evident that clinical research in this field is still in its infancy and, as yet, has not provided enough evidence to give a reliable response regarding the effectiveness of this class of intervention, consistent with existing narrative reviews of selected CBM programs or targeting one particular addictive behavior (Christiansen et al. 2015; Mühlig et al. 2017; Wiers et al. 2018). Further, training protocols of CBM are not consistent, with different amounts of sessions and trials per sessions, inclusion of filler trials mixed with training trials, different instructions or training task parameters (e.g., stimulus onset asynchrony), different intervention settings, and so on. These differences across training programs can create problems in both the inclusion and comparisons across studies since it would imply the addition of too many study-level moderators to adequately model sources of variance other than the primary therapeutic effects, for which there are not enough observations. In fact, we could not include one of the planned moderators, that is, training setting, in the analyses, due to the inclusion of only three studies conducted in an unsupervised environment (Elfeddali et al. 2016; Wiers et al. 2015b; Wittekind et al. 2015).

It is then crucial for the successful reproducibility of results and advance in the accumulation of evidence on the clinical efficacy of CBM to 1) endorse a systematic design and reporting of results of CBM behavior change studies as for other treatment interventions (e.g., CONSORT guidelines, Boutron et al. 2008, 2017), and including measures of training adherence as part of intervention outcomes; 2) share CBM intervention protocols and systematically test any change to procedure or contents before deploying such protocols into full treatment programs, since more studies are necessary to ensure the reproducibility of robust effects of the *same* treatment protocol; 3) carefully consider the selection of the intervention comparison (i.e., control condition) based on the research question at hand, and 4) increase the study quality, since despite the overall quality being generally high, some methodological issues in the studies included in this meta-analysis are likely to have introduced sources of bias. These issues related particularly to the generation of the randomization sequence and the related concealment of treatment allocation, which were not applied or guaranteed in almost half of the included studies. Robust methods of randomization in trials are essential to minimize allocation and selection bias and are technically easy to implement in the case of computerized interventions such as CBM training programs, since software used to implement and deliver the training program can also automatically randomize participants independently from the study personnel, who are then kept fully blinded to the assigned conditions and cannot predict the next participant assignment.

A last limitation affecting the quantitative aggregated analysis is the dependency between some of the included comparisons. Some studies included non-independent observations, for example by using several measures for the same outcome (i.e., Begh et al., 2015; Eberl et al. 2013; Schoenmakers et al. 2010; Wiers et al. 2011) or contrasting multiple CBM interventions to the same control condition (Wiers et al. 2015b). This can be problematic, since our statistical tools assume that analyses are conducted with an independent set of observations, that is, the value of one observation is not meant to be affected by the value of another, and that the effect sizes are independent realizations from a single overarching distribution. However, in our case, this is unlikely to have any serious consequences for our conclusions, since the independence of the observations (or more precisely, of residuals) has the effect of increasing the risk of error of Type I, namely, to reject the null hypothesis wrongly. However, more attention should be paid in future research to the independence of observations within the same study (i.e., include a within- or a between-subjects design with a single measure for each outcome).

In conclusion, the results of this meta-analysis confirmed the absence of enough evidence either in favor of or against of CBM as a behavior change intervention in alcohol use disorders and tobacco use disorders. However, based on the limited existing evidence, CBM has shown a modest impact on reducing the targeted cognitive bias(es), and, when the goal of the treatment was full abstinence, some indication of a small effect on the chance of relapse at follow-up, although with paradoxical effects with increased training practice. No other study- or participant-level moderator affected the impact of CBM on the outcomes. The included studies mainly focused on testing whether CBM works as a treatment intervention, and not if and under which circumstances, which would be more in line with an experimental medicine approach (Sheeran et al. 2017; Wiers et al. 2018). Indeed, only one study tested a moderated mediation (Eberl et al. 2013) to evaluate the mechanisms of change of the observed clinical effects, that is, whether the change in the behavioral outcome was a result of the change in the cognitive process targeted by the training intervention, and for whom this occurred. Very little can be said regarding whether CBM does impact addictive behaviors through changing the targeted dysfunctional information processing of appetitive cues in the environment, or if other non-specific components of CBM paradigms have an effect, which would also appear when using a sham version of the training.

Therefore, we do not suggest stopping investigating CBM as a behavior change intervention, as more evidence is necessary to reach a valid and unequivocal conclusion. However, we strongly recommend a careful reappraisal of choices in experimental design and methodology in the shift from the proof-of-principle, fundamental phase of research on the mechanisms at work in CBM, to the establishment of its clinical efficacy. Clinical efficacy studies naturally address different research questions, thus involving different choices in terms of study design, but also need to adhere to a stricter array of methodological standards, as to also efficiently allow for an integrative synthesis of the available evidence.

## Electronic supplementary material

ESM 1 (DOCX 105 kb)

## Footnotes

1 All studies used the Approach Avoidance Task task to deliver the training. In Wiers et al. (2015a, b), one version of the Approach Bias Modification explicitly instructed participants to react to alcohol-related and non-alcohol-related stimuli, while the other two varieties implicitly instructed participants to react to the stimulus format in 90 and 100% of the trials, respectively. In ITT analyses, the three Approach Bias Modification conditions did not show any significant difference in the effect on drinking reduction at follow-up. Therefore, they were merged together for the current meta-analysis similar to Wiers et al. (2011), where no difference in training effects was found between explicit and implicit instructions. Similarly, in Wittekind et al. (2015), an additional Approach Bias Modification condition presented an adjusted version of the Approach Avoidance Task training presenting RT feedback at the end of each trial. Although this version underperformed the standard training in per-protocol analyses, both of them showed a significant reduction in the substance use outcome at follow-up in the ITT results.

2 Due to the small amount of observations, training setting (supervised, such as clinic or research lab, and unsupervised, such as online) was not included as a moderator in the models. The large majority of studies were conducted in supervised settings, providing too few observations to evaluate moderation effects of setting (i.e., very small variance in the moderator).
