# Supplementary material for: Cognitive Bias Modification for Behavior Change in Alcohol and Smoking Addiction: Bayesian Meta-Analysis of Individual Participant Data
Source: Neuropsychol Rev. 2019 Jan 14;29(1):52–78. doi: 10.1007/s11065-018-9386-4 (PMC6499757; doi:10.1007/s11065-018-9386-4)
Supplement: Supplementary file 1 — (DOCX 105 kb) [file 11065_2018_9386_MOESM1_ESM.docx]

**SUPPLEMENTARY MATERIAL**

**Cognitive Bias Modification for behavior change in alcohol and smoking addiction: Bayesian meta-analysis of individual participant data**

Marilisa Boffo^a^, Oulmann Zerhouni^b^, Quentin F. Gronau^a^, Ruben J.J. van Beek^c^, Kyriaki Nikolaou^d^, Maarten Marsman^a^ and Reinout W. Wiers^a,1^

^a^ Department of Psychology, University of Amsterdam, NL

^b^ Department of Psychology, University Paris Nanterre, Paris, FR

^c^ Trimbos Instituut, Netherlands Institute of Mental Health and Addiction, Utrecht, NL

^d^ School of Psychology, University of Sussex, Falmer, UK

Correspondence concerning this article should be addressed to

Dr. Marilisa Boffo and Prof. Reinout W. Wiers,

Dept. of Psychology, University of Amsterdam

Nieuwe Achtergracht 129B, 1018 WS Amsterdam

Email: [marilisa.boffo@gmail.com](mailto:m.boffo@uva.nl) / [r.w.wiers@gmail.com](mailto:r.w.wiers@gmail.com)

Mail Address: Postbus 15916, 1001 NK Amsterdam

**Search Strings and results for each databases**

**Databases**

PsycINFO   976 results (May 18, 2016)

Medline   904 results (May 18, 2016)

Web of Science 1.262 results (May 18, 2016)

Embase 1.444 results (May 18, 2016)

Cochrane library   269 results (May 18, 2016)

Total 4.854 results

Total, de-duplicated 2.579 results

**PsycINFO**

*Ovid*

**#1 cognitive bias modification interventions**

cognitive bias/ OR attentional bias/ OR selective attention/ OR approach avoidance/ OR approach behavior/ OR response inhibition/ OR cognitive control/ OR (((cognitive or memory or attention*) adj3 bias*) OR selective attention OR approach bias* OR avoid* bias* OR (approach* ADJ3 tendenc*) OR approach-avoidance OR CBM intervention* OR CBM training* OR implicit association* OR evaluative conditioning OR inhibitory control* OR inhibition task* OR inhibitory process* OR response inhibition* OR (attention* ADJ1 (retraining OR training OR modification))).ti,ab,id,tm.

**Results: 32.128**

**#2 Addiction (smoking or alcohol)**

alcoholism/ OR alcohol abuse/ OR alcohol drinking patterns/ OR drinking behavior/ OR alcohol intoxication/ OR smoking cessation/ OR tobacco smoking/ OR nicotine/ OR nicotine withdrawal/ OR (alcohol* OR binge drink* OR drug* OR heavy drink* OR nicotin* OR smoking OR smoker* OR tobacco OR cigaret* OR drinker* OR drinking behavio*).ti,ab,id.

**Results: 314.813**

**#3 intervention**

(followup study OR longitudinal study OR systematic review OR meta analysis OR "treatment outcome/clinical trial").md. OR brain training/ OR training/ OR computer training/ OR followup studies/ OR intervention/ OR longitudinal studies/ OR treatment effectiveness evaluation/ OR treatment outcomes/ OR online therapy/ OR computer assisted therapy/ OR stroop color word test/ OR stroop effect/ OR (follow up OR followup OR intervention* OR longitudinal* OR training OR retraining OR re-training OR posttraining OR implicit association* test* OR implicit association* task* OR IAT OR probe* task* OR probe* test*  OR probe* experiment OR probe* paradigm OR priming task* OR priming test* OR approach avoidance task* OR approach avoidance test* OR AAT OR stroop OR gonogo OR go-nogo OR go-no-go OR AACTP OR SRC OR stimulus-response compatibility OR visual search task* OR visual search test*).ti,ab,id. OR (approach avoidance OR nogo OR no go).tm.

**Results: 749.547**

**#4 animal**

animal.po

**1 AND 2 AND 3 1.056 results**

**NOT 4   979 results**

**Medline***Epub Ahead of Print, In-Process & Other Non-Indexed Citations, Ovid MEDLINE(R) Daily and Ovid MEDLINE(R) 1946 to 18 May 2016*

**#1 cognitive bias modification interventions**

avoidance learning/ OR "inhibition (psychology)"/ OR (((cognitive or memory or attention*) ADJ3 bias*) OR selective attention OR approach bias* OR avoid* bias* OR (approach* ADJ3 tendenc*) OR approach-avoidance OR CBM intervention* OR CBM training* OR implicit association* OR evaluative conditioning OR inhibitory control* OR inhibition task* OR inhibitory process* OR response inhibition* OR (attention* ADJ1 (retraining OR training OR modification))).ti,ab,kf.

**Results: 46.447**

**#2 Addiction (smoking or alcohol)**

alcoholism/ OR alcohol drinking/ OR binge drinking/ OR drinking behavior/ OR nicotine/ OR smoking/ OR (alcohol* OR binge drink* OR drug* OR heavy drink* OR nicotin* OR smoking OR smoker* OR tobacco OR cigaret* OR drinker* OR drinking behavio*).ti,ab,kf.

**Results: 1.850.861**

**#3 intervention**

follow-up studies/ OR longitudinal studies/ OR treatment outcome/ OR therapy, computer-assisted/ OR stroop test/ OR (follow up OR followup OR intervention* OR longitudinal* OR training OR retraining OR re-training OR posttraining OR implicit association* test* OR implicit association* task* OR IAT OR probe* task* OR probe* test*  OR probe* experiment OR probe* paradigm OR priming task* OR priming test* OR approach avoidance task* OR approach avoidance test* OR AAT OR stroop OR gonogo OR go-nogo OR go-no-go OR AACTP OR SRC OR stimulus-response compatibility OR visual search task* OR visual search test*).ti,ab,kf.

**Results: 2.525.337**

**#4 animals**

animals/ OR exp rodentia/

**1 AND 2 AND 3 1.663 results**

**NOT 4   904 results**

**Web of Science**

**#1 cognitive bias modification interventions**

TS=((("cognitive" OR "memory" OR "attention*") NEAR/2 "bias*") OR "selective attention" OR "approach bias*" OR "avoid* bias*" OR ("approach*" NEAR/2 "tendenc*") OR "approach-avoidance" OR "CBM intervention*" OR "CBM training*" OR "implicit association*" OR "evaluative conditioning" OR "inhibitory control*" OR "inhibition task*" OR "inhibitory process*" OR "response inhibition*" OR ("attention*" NEAR/0 ("retraining" OR "training" OR "modification")))

**Results: 36.047**

**#2 Addiction (smoking or alcohol)**

TS=("alcohol*" OR "binge drink*" OR "drug*" OR "heavy drink*" OR "nicotin*" OR "smoking" OR "smoker*" OR "tobacco" OR "cigaret*" OR "drinker*" OR "drinking behavio*")

**Results: 1.898.957**

**#3 intervention**

TS=("follow up" OR "followup" OR "intervention*" OR "longitudinal*" OR "training" OR "retraining" OR "re-training" OR "posttraining" OR "implicit association* test*" OR "implicit association* task*" OR "IAT" OR "probe* task*" OR "probe* test* " OR "probe* experiment" OR "probe* paradigm" OR "priming task*" OR "priming test*" OR "approach avoidance task*" OR "approach avoidance test*" OR "AAT" OR "stroop" OR "gonogo" OR "go-nogo" OR "go-no-go" OR "AACTP" OR "SRC" OR "stimulus-response compatibility" OR "visual search task*" OR "visual search test*")

**Results: 1.905.167**

**1 AND 2 AND 3 1.262 results**

**Embase**

*Ovid, Embase Classic+Embase 1947 to 2016 (May 18)*

**#1 cognitive bias modification interventions**

cognitive bias/ OR attentional bias/ OR attentional retraining/ OR avoidance behavior/ OR "inhibition (psychology)"/ OR (((cognitive or memory or attention*) adj3 bias*) OR selective attention OR approach bias* OR avoid* bias* OR (approach* ADJ3 tendenc*) OR approach-avoidance OR CBM intervention* OR CBM training* OR implicit association* OR evaluative conditioning OR inhibitory control* OR inhibition task* OR inhibitory process* OR response inhibition* OR (attention* ADJ1 (retraining OR training OR modification))).ti,ab,kw.

**Results: 54.885**

**#2 Addiction (smoking or alcohol)**

alcoholism/ OR alcohol abuse/ OR alcohol consumption/ OR drinking behavior/ OR alcohol intoxication/ OR smoking cessation/ OR cigarette smoking/ OR nicotine/ OR (alcohol* OR binge drink* OR drug* OR heavy drink* OR nicotin* OR smoking OR smoker* OR tobacco OR cigaret* OR drinker* OR drinking behavio*).ti,ab,kw.

**Results: 2.564.893**

**#3 intervention**

training/ OR follow up/ OR intervention study/ OR longitudinal study/ OR computer assisted therapy/ OR stroop test/ OR action tendency training/ OR alcohol attention control training program/ OR visual probe task/ OR (follow up OR followup OR intervention* OR longitudinal* OR training OR retraining OR re-training OR posttraining OR implicit association* test* OR implicit association* task* OR IAT OR probe* task* OR probe* test*  OR probe* experiment OR probe* paradigm OR priming task* OR priming test* OR approach avoidance task* OR approach avoidance test* OR AAT OR stroop OR gonogo OR go-nogo OR go-no-go OR AACTP OR SRC OR stimulus-response compatibility OR visual search task* OR visual search test*).ti,ab,kw.

**Results: 2.772.629**

**#4 animal**

animal/ OR animal experiment/ OR animal model/ OR nonhuman/

**1 AND 2 AND 3 2.166 results**

**NOT 4 1.592 results**

**NOT Medline 1.444 results**

**Cochrane Library**

**#1 cognitive bias modification interventions**

(("cognitive":ti,ab,kw OR "memory":ti,ab,kw OR "attention*":ti,ab,kw) NEAR/2 "bias*":ti,ab,kw) OR "selective attention":ti,ab,kw OR "approach bias*":ti,ab,kw OR "avoid* bias*":ti,ab,kw OR ("approach*":ti,ab,kw NEAR/2 "tendenc*":ti,ab,kw) OR "approach-avoidance":ti,ab,kw OR "CBM intervention*":ti,ab,kw OR "CBM training*":ti,ab,kw OR "implicit association*":ti,ab,kw OR "evaluative conditioning":ti,ab,kw OR "inhibitory control*":ti,ab,kw OR "inhibition task*":ti,ab,kw OR "inhibitory process*":ti,ab,kw OR "response inhibition*":ti,ab,kw OR ("attention*":ti,ab,kw NEAR/0 ("retraining":ti,ab,kw OR "training":ti,ab,kw OR "modification":ti,ab,kw))

**Results: 1.813**

**#2 Addiction (smoking or alcohol)**

"alcohol*":ti,ab,kw OR "binge drink*":ti,ab,kw OR "drug*":ti,ab,kw OR "heavy drink*":ti,ab,kw OR "nicotin*":ti,ab,kw OR "smoking":ti,ab,kw OR "smoker*":ti,ab,kw OR "tobacco":ti,ab,kw OR "cigaret*":ti,ab,kw OR "drinker*":ti,ab,kw OR "drinking behavio*":ti,ab,kw

**Results: 295.037**

**#3 intervention**

"follow up":ti,ab,kw OR "followup":ti,ab,kw OR "intervention*":ti,ab,kw OR "longitudinal*":ti,ab,kw OR "training":ti,ab,kw OR "retraining":ti,ab,kw OR "re-training":ti,ab,kw OR "posttraining":ti,ab,kw OR "implicit association* test*":ti,ab,kw OR "implicit association* task*":ti,ab,kw OR "IAT":ti,ab,kw OR "probe* task*":ti,ab,kw OR "probe* test* ":ti,ab,kw OR "probe* experiment":ti,ab,kw OR "probe* paradigm":ti,ab,kw OR "priming task*":ti,ab,kw OR "priming test*":ti,ab,kw OR "approach avoidance task*":ti,ab,kw OR "approach avoidance test*":ti,ab,kw OR "AAT":ti,ab,kw OR "stroop":ti,ab,kw OR "gonogo":ti,ab,kw OR "go-nogo":ti,ab,kw OR "go-no-go":ti,ab,kw OR "AACTP":ti,ab,kw OR "SRC":ti,ab,kw OR "stimulus-response compatibility":ti,ab,kw OR "visual search task*":ti,ab,kw OR "visual search test*":ti,ab,kw

**Results: 245.409**

**1 AND 2 AND 3 269 results**

**Reference list of included studies**

Begh, R., Munafò, M. R., Shiffman, S., Ferguson, S. G., Nichols, L., Mohammed, M. A., et al. (2015). Lack of attentional retraining effects in cigarette smokers attempting cessation: A proof of concept double-blind randomised controlled trial. Drug and Alcohol Dependence, 149, 158-165.

Clerkin, E. M., Magee, J. C., Wells, T. T., Beard, C., & Barnett, N. P. (2016). Randomized controlled trial of attention bias modification in a racially diverse, socially anxious, alcohol dependent sample. Behaviour Research and Therapy, 87, 58-69.

Cox, W. M., Fadardi, J. S., Hosier, S. G., & Pothos, E. M. (2015). Differential effects and temporal course of attentional and motivational training on excessive drinking. Experimental and Clinical Psychopharmacology, 23(6), 445.

Eberl, C., Wiers, R. W., Pawelczack, S., Rinck, M., Becker, E. S., & Lindenmeyer, J. (2013). Approach bias modification in alcohol dependence: do clinical effects replicate and for whom does it work best? Developmental Cognitive Neuroscience, 4, 38-51.

Elfeddali, I., de Vries, H., Bolman, C., Pronk, T., & Wiers, R. W. (2016). A randomized controlled trial of Web-based Attentional Bias Modification to help smokers quit. Health Psychology, 35(8), 870-880.

Kong, G., Larsen, H., Cavallo, D. A., Becker, D., Cousijn, J., Salemink, E., Collot D’Escury- Koenigs, A. L., Morean, M. E., Wiers, R. W., & Krishnan-Sarin, S. (2015). Re-training automatic action tendencies to approach cigarettes among adolescent smokers: a pilot study. The American Journal of Drug and Alcohol Abuse, 41(5), 425-432.

Lopes, F. M., Pires, A. V., & Bizarro, L. (2014). Attentional bias modification in smokers trying to quit: A longitudinal study about the effects of number of sessions. Journal of Substance Abuse Treatment, 47(1), 50-57.

Machulska, A., Zlomuzica, A., Rinck, M., Assion, H. J., & Margraf, J. (2016). Approach bias modification in inpatient psychiatric smokers. Journal of Psychiatric Research, 76, 44- 51.

McHugh, R. K., Murray, H. W., Hearon, B. A., Calkins, A. W., & Otto, M. W. (2010). Attentional bias and craving in smokers: the impact of a single attentional training session. Nicotine & Tobacco Research, 12(12), 1261-1264.

Schoenmakers, T. M., de Bruin, M., Lux, I. F., Goertz, A. G., Van Kerkhof, D. H., & Wiers, R. W. (2010). Clinical effectiveness of attentional bias modification training in abstinent alcoholic patients. Drug and Alcohol Dependence, 109(1), 30-36.

Wiers, C. E., Ludwig, V. U., Gladwin, T. E., Park, S. Q., Heinz, A., Wiers, R. W., et al. (2015). Effects of cognitive bias modification training on neural signatures of alcohol approach tendencies in male alcohol‐dependent patients. Addiction Biology, 20(5), 990-999.

Wiers, R. W., Eberl, C., Rinck, M., Becker, E. S., & Lindenmeyer, J. (2011). Retraining automatic action tendencies changes alcoholic patients’ approach bias for alcohol and improves treatment outcome. Psychological Science, 22(4), 490-497.

Wiers, R. W., Houben, K., Fadardi, J. S., van Beek, P., Rhemtulla, M., & Cox, W. M. (2015). Alcohol cognitive bias modification training for problem drinkers over the web. Addictive Behaviors, 40, 21-26.

Wittekind, C. E., Feist, A., Schneider, B. C., Moritz, S., & Fritzsche, A. (2015). The approach-avoidance task as an online intervention in cigarette smoking: a pilot study. Journal of Behavior Therapy and Experimental Psychiatry, 46, 115-120.

**One-stage Bayesian IPD meta-analysis**

Table S1. Results of the hierarchical mixed-effects models $M_{0}$ to $M_{6}$ for the cognitive bias outcome in the Bayesian 1-stage IPD meta-analysis (95% credible interval between brackets).

|  | $\boldsymbol{M}_{\boldsymbol{0}}$ | $\boldsymbol{M}_{\boldsymbol{1}}$ | $\boldsymbol{M}_{\boldsymbol{2}}$ | $\boldsymbol{M}_{\boldsymbol{3}}$ | $\boldsymbol{M}_{\boldsymbol{4}}$ | $\boldsymbol{M}_{\boldsymbol{5}}$ | $\boldsymbol{M}_{\boldsymbol{6}}$ |
| --- | --- | --- | --- | --- | --- | --- | --- |
| $\theta$ | 0.23 (0.06, 0.41) | 0.25 (0.08, 0.42) | 0.24 (0.09, 0.39) | 0.24 (0.08, 0.40) | 0.22 (0.02, 0.42) | 0.24 (0.06, 0.43) | 0.22 (0.03, 0.42) |
| $\tau^{2}$ | 0.09 (0.02, 0.25) | 0.08 (0.02, 0.23) | 0.05 (0.01, 0.16) | 0.06 (0.01, 0.18) | 0.07 (0.01, 0.22) | 0.04 (0.00, 0.17) | 0.05 (0.00, 0.19) |
| Duration of follow-up |  | -0.11 (-0.23, 0.03) | -0.08 (-0.20, 0.04) | -0.07 (-0.20, 0.06) | -0.06 (-0.20, 0.09) | -0.06 (-0.20, 0.07) | -0.06 (-0.20, 0.07) |
| Addiction type |  |  | -0.17 (-0.33, -0.02) | -0.20 (-0.39, 0.00) | -0.20 (-0.41, -0.00) | -0.22 (-0.40, -0.03) | -0.24 (-0.43, -0.04) |
| Type of CBM training |  |  |  | -0.04 (-0.23, 0.15) | -0.05 (-0.26, 0.14) | -0.01 (-0.21, 0.17) | -0.04 (-0.24, 0.16) |
| Addiction type * Type of CBM training |  |  |  |  | -0.04 (-0.24, 0.17) | -0.06 (-0.24, 0.13) | -0.05 (-0.24, 0.16) |
| No. training trials |  |  |  |  |  | -0.07 (-0.17, 0.04) | -0.03 (-0.18, 0.13) |
| No. training trials * Condition |  |  |  |  |  |  | -0.06 (-0.21, 0.10) |
| $\mathrm{logBF}_{i0}$ | ­– | -1.60 | -1.79 | -4.32 | -6.77 | -8.96 | -11.47 |

Table S2. Results for the hierarchical mixed-effects model $M_{7}$ on the reduced dataset for the cognitive bias outcome in the Bayesian 1-stage IPD meta-analysis (95% credible interval between brackets).

|  | $\boldsymbol{M}_{\boldsymbol{7}}$ |
| --- | --- |
| $\theta$ | 0.10 (-0.11, 0.31) |
| $\tau^{2}$ | 0.03 (0.00, 0.14) |
| Duration of follow-up | -0.06 (-0.19, 0.06) |
| Addiction type | -0.13 (-0.33, 0.07) |
| Type of CBM training | 0.07 (-0.13, 0.28) |
| Addiction type * Type of CBM Training | -0.16 (-0.35, 0.05) |
| No. Training trials | -0.01 (-0.16, 0.14) |
| No. Training trials * Condition | -0.09 (-0.25, 0.07) |
| Severity of substance use | -0.01 (-0.06, 0.03) |
| $\log\mathrm{BF}_{70}$ | -14.11 |

Table S3. Results for the hierarchical mixed-effects models $M_{0}$ to $M_{6}$ for the reduction of substance use outcome in the Bayesian 1-stage IPD meta-analysis (95% credible interval between brackets)

|  | $\boldsymbol{M}_{\boldsymbol{0}}$ | $\boldsymbol{M}_{\boldsymbol{1}}$ | | $\boldsymbol{M}_{\boldsymbol{2}}$ | | $\boldsymbol{M}_{\boldsymbol{3}}$ | | $\boldsymbol{M}_{\boldsymbol{4}}$ | | $\boldsymbol{M}_{\boldsymbol{5}}$ | | $\boldsymbol{M}_{\boldsymbol{6}}$ | |
| --- | --- | --- | --- | --- | --- | --- | --- | --- | --- | --- | --- | --- | --- |
| $\theta$ | 0.19 (-0.23, 0.58) | | 0.19 (-0.32, 0.65) | | 0.18 (-0.39, 0.69) | | 0.16 (-0.54, 0.81) | | 0.09 (-0.73, 0.94) | | 0.07 (-0.75, 0.91) | | 0.07 (-0.77, 0.96) |
| $\tau^{2}$ | 0.22 (0.01, 1.22) | | 0.35 (0.01, 2.05) | | 0.45 (0.00, 2.86) | | 0.77 (0.01, 4.29) | | 1.06 (0.01, 5.61) | | 1.07 (0.01, 5.62) | | 1.19 (0.01, 6.12) |
| Duration of follow-up |  | | -0.02 (-0.38, 0.36) | | -0.04 (-0.47, 0.40) | | 0.01 (-0.63, 0.66) | | 0.08 (-0.78, 0.92) | | 0.06 (-0.83, 0.85) | | 0.05 (-0.85, 0.88) |
| Addiction type |  | |  | | 0.15 (-0.41, 0.67) | | 0.09 (-0.65, 0.83) | | 0.04(-0.85, 0.93) | | 0.02 (-0.83, 0.91) | | -0.20 (-1.16, 0.80) |
| Type of CBM training |  | |  | |  | | 0.10 (-0.71, 0.92) | | 0.16 (-0.85, 1.12) | | 0.19 (-0.85, 1.12) | | 0.06 (-1.00, 1.07) |
| Addiction type * Type of CBM training |  | |  | |  | |  | | 0.11 (-0.79, 0.95) | | 0.13 (-0.81, 0.99) | | 0.26 (-0.70, 1.18) |
| No. Training trials |  | |  | |  | |  | |  | | 0.28 (-0.08, 0.63) | | 0.36 (-0.03, 0.76) |
| No. Training trials * Condition |  | |  | |  | |  | |  | |  | | -0.18(-0.53, 0.15) |
| $\log\mathrm{BF}_{i0}$ | – | | -2.14 | | -3.46 | | -4.80 | | -5.99 | | -6.76 | | -8.19 |

Table S4. Results for the hierarchical mixed-effects model $M_{7}$ on the reduced dataset for the reduction of substance use outcome in the Bayesian 1-stage IPD meta-analysis (95% credible interval between brackets)

|  | $\boldsymbol{M}_{\boldsymbol{7}}$ |
| --- | --- |
| $\theta$ | 0.05 (-0.80, 0.94) |
| $\tau^{2}$ | 1.27 (0.01, 6.49) |
| Duration of follow-Up | 0.05 (-0.88, 0.88) |
| Addiction type | -0.21 (-1.18, 0.78) |
| Type of CBM Training | 0.03 (-1.09, 0.99) |
| Addiction type * Type of CBM training | 0.29 (-0.73, 1.23) |
| No. Training trials | 0.34 (-0.04, 0.74) |
| No. Training trials * Condition | -0.21 (-0.56, 0.12) |
| Severity of substance use | 0.18 (0.11, 0.26) |
| $\log\mathrm{BF}_{70}$ | -0.11 |

Table S5. Results for the hierarchical mixed-effects logistic models $M_{0}$ to $M_{6}$ for the relapse outcome in the Bayesian 1-stage IPD meta-analysis (effects are expressed in log odds; 95% central credible interval between brackets)

|  | $\boldsymbol{M}_{\boldsymbol{0}}$ | | $\boldsymbol{M}_{\boldsymbol{1}}$ | | $\boldsymbol{M}_{\boldsymbol{2}}$ | | $\boldsymbol{M}_{\boldsymbol{3}}$ | | $\boldsymbol{M}_{\boldsymbol{4}}$ | | $\boldsymbol{M}_{\boldsymbol{5}}$ | | $\boldsymbol{M}_{\boldsymbol{6}}$ | |
| --- | --- | --- | --- | --- | --- | --- | --- | --- | --- | --- | --- | --- | --- | --- |
| $\theta$ | | -0.27 (-0.68, 0.22) | | -0.25 (-0.71, 0.30) | | -0.24 (-0.74, 0.35) | | -0.24 (-0.77, 0.38) | | 0.00(-0.90, 0.90) | | 0.14 (-0.89, 1.20) | | 0.26 (-0.97, 1.55) |
| $\tau^{2}$ | | 0.21 (0.01, 1.15) | | 0.33 (0.00, 1.82) | | 0.43 (0.01, 2.32) | | 0.49 (0.01, 2.62) | | 0.52 (0.01, 2.98) | | 1.36 (0.09, 5.50) | | 2.81 (0.29, 9.48) |
| Duration of follow-up | |  | | 0.02(-0.43, 0.47) | | 0.03 (-0.51, 0.58) | | 0.11 (-0.56, 0.75) | | 0.22(-0.53, 0.95) | | 0.22(-0.72, 1.17) | | 0.30 (-0.80, 1.46) |
| Addiction type | |  | |  | | 0.01 (-0.61, 0.64) | | -0.10 (-0.85, 0.67) | | 0.02(-0.83, 0.87) | | -0.09 (-1.11, 0.91) | | 0.19 (-0.96, 1.46) |
| Type of CBM training | |  | |  | |  | | -0.21 (-1.08, 0.65) | | -0.17 (-1.09, 0.70) | | -0.03 (-1.08, 1.03) | | 0.04 (-1.20, 1.30) |
| Addiction type *  Type of CBM training | |  | |  | |  | |  | | 0.31(-0.61, 1.24) | | 0.13 (-0.95, 1.23) | | -0.16 (-1.48, 1.06) |
| No. Training trials | |  | |  | |  | |  | |  | | -0.56 (-0.93, -0.22) | | -1.29 (-1.98, -0.66) |
| No. Training trials *  Condition | |  | |  | |  | |  | |  | |  | | 0.87 (0.27, 1.54) |
| $\mathrm{logBF}_{i0}$ | | – | | -1.87 | | -3.36 | | -4.33 | | -5.14 | | -1.36 | | 1.47 |

Table S6. Results for the hierarchical mixed-effects logistic model $M_{7}$ on the reduced dataset for the relapse outcome in the Bayesian 1-stage IPD meta-analysis (effects are expressed in log odds; 95% central credible interval between brackets).

|  | $\boldsymbol{M}_{\boldsymbol{7}}$ |
| --- | --- |
| $\theta$ | 0.28 (-1.21, 1.94) |
| $\tau^{2}$ | 3.12 (0.32, 10.65) |
| Duration of follow-up | 0.27 (-0.99, 1.59) |
| Addiction type | 0.17 (-1.51, 1.98) |
| Type of CBM training | 0.02 (-1.57, 1.66) |
| Addiction type * Type of CBM Training | -0.13 (-1.77, 1.43) |
| No. Training trials | -1.27 (-1.97, -0.64) |
| No. Training trials * Condition | 0.83 (0.23, 1.51) |
| Severity of substance use | 0.13 (-0.01, 0.26) |
| $\mathrm{logBF}_{70}$ | 0.67 |

**One-stage Frequentist IPD meta-analysis**

**Data analysis**

All analyses were conducted in R (R Core Team, 2017) using the *lme4* and *LmerTest* packages and the *lmer* and *glmer* functions. We calculated the standardized $\text{β}$ coefficient (and odds ratio, OR, for relapse rate) for the examined comparisons. This estimate indicates how many SDs the dependent variable (difference in cognitive bias score and amount of drinks or cigarettes/week between baseline and follow-up, or the OR of relapse) changes per SD increase in the predictor variable. A positive $\text{β}$ coefficient indicates a larger reduction in cognitive bias and substance consumption, while an OR > 1.00 a relative higher risk of relapse. Alcohol use disorder and AtBM training were coded -1 and tobacco use disorder and ApBM training +1. Control and training condition were coded -1 and +1, respectively.

For all outcomes we used the same modeling approach as in the Bayesian analyses. For the cognitive bias and reduction in substance use outcomes, we used a multilevel mixed-effects linear regression to estimate the same models (i.e., $M_{0}$ to $M_{7}$), with the difference in the outcome scores between baseline and follow-up as dependent variable. All models included a random study effect (i.e., random intercept) to control for unobserved study heterogeneity. IPD data was considered level 1 and study-level data was considered level 2. At level 1, all models included a fixed effect with random intercept and slope for training condition (i.e., model $M_{1}$). However, because models tended to be overidentified (i.e., perfect random slope-intercept correlation), we used the *optimx* package with a Limited-Memory BFGS algorithm to optimize the estimation of random effects. Following the recommendation from Barr et al. (2013), we also computed simplified models without the estimation of a random intercept parameter for condition. In both cases, estimates of fixed effects and random effects were not significantly affected. For simplicity, we reported only the modes including both the random intercept and random slope for condition. The R scripts available at <https://osf.io/dbcsz/> provide the code to compute both sets of models.

For the relapse outcome, we used a multi-level mixed-effects logistic regression including a fixed effect and random intercept and slope for training condition at level 1. In this case, the effect of training condition would be observed in a significant OR coefficient > 1 (i.e., greater chance of relapse for the control compared to training condition).

On top of the basic model $M_{0}$ including the effect of condition, seven statistical models (model $M_{1}$ to $M_{7}$) were further computed for each outcome, progressively including fixed effects for covariates at level 2 (i.e., duration of follow-up, targeted addiction disorder and type of CBM training) and level 1 (i.e., amount of training trials and severity of substance use problems), and for the specified interactions. We did not compute the null model (i.e., the intercept) in the frequentist analyses since it does not provide any relevant insight into the effects of the included variable in model $M_{0}$.

We computed the AIC index for each model and the difference in AIC (${\Delta AIC}_{Mi}$) between a target model *i* and the model with the lowest AIC value (i.e., the best fitted model) to determine which model was most parsimonious and better fitting the data for each outcome (Burnham et al., 2011). As a rule of thumb, a ${\Delta AIC}_{Mi}$ ≤ 2 for model M*i* suggests substantial evidence for the model, values between 3 and 7 indicate that the model has considerably less support, whereas a ${\Delta AIC}_{i}$ > 10 indicates that the model is very unlikely (Burnham & Anderson, 2002, p.70).

**Results**

**Change in cognitive bias.** Table S7 includes the main findings of the IPD meta-analysis on cognitive bias. Overall, AIC and ${\Delta AIC}_{Mi}$ showed model $M_{7}$ to best fit to the data (AIC = 5692, ${\Delta AIC}_{M7-M7}$ = 0), while the second best fitting model ($M_{0}$) showed a relatively poor fit (AIC = 5948, ${\Delta AIC}_{M0-M7}$ = 256). In all models the main effect of training condition was significant and became stronger once all moderators were included in the final model $M_{7}$ ($\beta$ = 0.14, *p* = .01), indicating a larger decrease in cognitive bias in the training compared to the control condition. However, none of the moderators added any significant main or moderating effect on cognitive bias in model $M_{7}$.

Note that in model $M_{7}$ two comparisons from the same study (Schoenmakers et al., 2010) where discarded from the analyses due to the lack of data on the severity of substance use problems covariate, making the comparison with the other models less unequivocal. When examining the goodness of fit of models including all comparisons, the simplest model $M_{0}$ only including training condition was the best fitting (AIC = 5948), with a positive effect of CBM on cognitive bias in the training relative to the control group ($\beta$ = 0.11, *p* = .004). Again, none of the moderators or covariates added any significant effect on the reduction in the targeted cognitive bias. Hence, overall, CBM seemed to reduce cognitive bias immediately after the end of the training intervention of about 0.1 standard deviations. Note that this effect was not affected by any study- or participant-level covariate or moderator.

Table S7. Hierarchical mixed-effects model results on changes in cognitive bias for the frequentist 1-stage IPD meta-analysis (96% confidence intervals between brackets)

|  | $\boldsymbol{M}_{\boldsymbol{0}}$ | | $\boldsymbol{M}_{\boldsymbol{1}}$ | | $\boldsymbol{M}_{\boldsymbol{2}}$ | | $\boldsymbol{M}_{\boldsymbol{3}}$ | | $\boldsymbol{M}_{\boldsymbol{4}}$ | | $\boldsymbol{M}_{\boldsymbol{5}}$ | | $\boldsymbol{M}_{\boldsymbol{6}}$ | | $\boldsymbol{M}_{\boldsymbol{7}}$ | |
| --- | --- | --- | --- | --- | --- | --- | --- | --- | --- | --- | --- | --- | --- | --- | --- | --- |
|  | **B** | **p** | **B** | **p** | **B** | **p** | **B** | **p** | **B** | **p** | **B** | **p** | **B** | **p** | **B** | **p** |
| **Fixed effects** |  |  |  |  |  |  |  |  |  |  |  |  |  |  |  |  |
| Intercept | -0.01 (-0.05, 0.03) | .683 | -0.01 (-0.05, 0.03) | .722 | -0.01 (-0.05, 0.03) | .735 | -0. 01 (-0.05, 0.03) | .735 | -0. 01 (-0.07, 0.05) | .814 | -0.00 (-0.15, 0.07) | .518 | -0.00 (-0.15, 0.07) | .517 | -0.00 (-0.19, 0.09) | .543 |
| Condition (training vs. control) | 0.11  (0.04, 0.18) | .004 | 0.11  (0.04, 0.18) | .004 | 0.11  (0.04, 0.18) | .004 | 0.11  (0.04, 0.18) | .004 | 0.11  (0.04, 0.18) | .004 | 0.10 (0.03, 0.17) | .007 | 0.10  (-0.01, 0.20) | .007 | 0.14 (0.03, 0.25) | .015 |
| Duration follow-up |  |  | -0.001 (-0.04, 0.04) | .972 | -0.001 (-0.04, 0.04) | .966 | -0.00 (-0.04, 0.04) | .960 | 0.00 (-0.04, 0.04) | .960 | -0.00  (-0.04, 0.04) | .961 | -0.0001  (-0.04, 0.04) | .980 | -0.001  (-0.05, 0.04) | .745 |
| Addiction type (tobacco vs. alcohol) |  |  |  |  | 0.00  (0.0, 0.04) | .956 | 0.00  (-0.05, 0.06) | .948 | 0. 00  (-0.05, 0.06) | .948 | 0.01  (-0.05, 0.06) | .808 | 0.01  (-0.05, 0.07) | .783 | 0.00  (-0.09, 0.09) | .902 |
| Type of CBM training (ApBM vs. AtBM) |  |  |  |  |  |  | 0.00  (-0.05, 0.05) | .971 | 0. 00  (-0.05, 0.06) | .971 | 0.01  (-0.05, 0.06) | .829 | 0.001  (-0.04, 0.04) | .871 | 0.02  (-0.04, 0.09) | .565 |
| Addiction type * Type of CBM training |  |  |  |  |  |  |  |  | 0. 00  (-0.04, 0.04) | .992 | -0.00  (-0.04, 0.04) | .902 | -0.00  (-0.04, 0.04) | .937 | -0.01  (-0.06, 0.04) | .826 |
| No. Training trials |  |  |  |  |  |  |  |  |  |  | .02  (-0.04,  0.08) | .531 | -.01  (-0.04, 0.08) | .555 | 0.03  (-0.02, 0.10) | .273 |
| Condition * No. Training trials |  |  |  |  |  |  |  |  |  |  |  |  | .007  (-0.09, 0.10) | .88 | -0.06  (-0.17, 0.03) | .272 |
| Severity substance use |  |  |  |  |  |  |  |  |  |  |  |  |  |  | -0.00  (-0.06, 0.06) | .986 |
| **Random components** |  |  |  |  |  |  |  |  |  |  |  |  |  |  |  |  |
| $\sigma^{2}$ | 0.966 | | 0.966 | | 0.966 | | 0.966 | | 0.966 | | 0.966 | | 0.966 | | 0.962 | |
| $\tau_{00, Study}$ | 0.000 | | 0.000 | | 0.000 | | 0.000 | | 0.000 | | 0.000 | | 0.000 | | 0.000 | |
| $\tau_{11, Study}$ | 0.010 | | 0.010 | | 0.010 | | 0.010 | | 0.010 | | 0.010 | | 0.010 | | 0.007 | |
| $\rho_{01}$ | -1.000 | | -1.000 | | -1.000 | | -1.000 | | -1.000 | | -1.000 | | -1.000 | | -1.000 | |
| N_Comparisons_ | 18 | | 18 | | 18 | | 18 | | 18 | | 18 | | 18 | | 16^a^ | |
| ICC_Study_ | 0.000 | | 0.000 | | 0.000 | | 0.000 | | 0.000 | | 0.000 | | 0.000 | | 0.000 | |
| Observations | 2112 | | 2112 | | 2112 | | 2112 | | 2112 | | 2112 | | 2112 | | 2020 | |
| ${R^{2}}/{\Omega_{0}^{2}}$ | .027/.027 | | .027/.027 | | .026/.026 | | .027/.026 | | .026/.026 | | .028/.026 | | .028/.030 | | .025/.024 | |
| AIC | 5948 | | 5950 | | 5952 | | 5954 | | 5956 | | 5957 | | 5959 | | 5692 | |

**^a^** Two comparisons were not included in model $M_{7}$ (Schoenmakers et al., 2010) due to the absence of IPD data on severity of substance use. B***:*** standardized beta coefficient; *95% C.I.:* 95% confidence interval for the standardized coefficient; *p****:*** p-value; $\sigma^{2}$: intra-study variance; $\tau_{00, Study}$: random intercept variance at the study level; $\tau_{11, Study}$: random slopes variance of condition at the study level; $\rho_{01}$: correlation between $\tau_{00, Study}$ and $\tau_{11, Study}$; *N_Comparisons_*: number of comparisons per model; *ICC_Study_*: intra-class correlation for studies; *Observations*: number of observation in each model; ${R^{2}}/{\Omega_{0}^{2}}$: R squared and Omega squared values for each model; *AIC*: Akaike Information Criterion.

**Reduction of substance use.** Table S8 includes the main findings of the IPD meta-analysis on substance use. Similar to cognitive bias, AIC and ${\Delta AIC}_{Mi}$ showed model $M_{7}$ to best fit to the data (AIC = 2186, ${\Delta AIC}_{M7-M7}$ = 0). However, the main effect of training condition was not significant ($\beta$ = .19; *p* = .12), nor in any of the other models. When looking at the main and interaction effects of the included moderators, a few effects were statistically significant: independently from condition, using ApBM training for tobacco, or AtBM training for alcohol, seems to have an better effect (greater decrease in consumption) ($\beta$ = 0.20, *p* = .04), alongside showing more severe substance use problems ($\beta$ = 0.21, *p* < .001).

In summary, we did not find any CBM-specific effect on the reduction in tobacco and alcohol consumption between baseline and follow-up (i.e., similar reduction across the training and control conditions).

Table S8. Hierarchical mixed-effects model results on reduction in substance use from baseline to follow-up for the frequentist 1-stage IPD meta-analysis (95% confidence intervals between brackets)

|  | $\boldsymbol{M}_{\boldsymbol{0}}$ | | $\boldsymbol{M}_{\boldsymbol{1}}$ | | $\boldsymbol{M}_{\boldsymbol{2}}$ | | $\boldsymbol{M}_{\boldsymbol{3}}$ | | $\boldsymbol{M}_{\boldsymbol{4}}$ | | $\boldsymbol{M}_{\boldsymbol{5}}$ | | $\boldsymbol{M}_{\boldsymbol{6}}$ | | $\boldsymbol{M}_{\boldsymbol{7}}$ | |
| --- | --- | --- | --- | --- | --- | --- | --- | --- | --- | --- | --- | --- | --- | --- | --- | --- |
|  | **B** | **p** | **B** | **p** | **B** | **p** | **B** | **p** | **B** | **p** | **B** | **p** | **B** | **p** | **B** | **p** |
| **Fixed effects** |  |  |  |  |  |  |  |  |  |  |  |  |  |  |  |  |
| Intercept | -0.02  (-0.10, 0.05) | .578 | -0.02  (-0.14, 0.09) | .713 | -0.02  (-0.13, 0.09) | .720 | -0.07  (-0.07, 0.22) | .358 | -0.01  (-0.27, 0.24) | .895 | -0.01  (-0.28, 0.25) | .914 | -0.08 (-0.25, 0.42) | .675 | -0.55 (-0.93, -0.17) | .004 |
| Condition (training vs. control) | .09  (-0.00, 0.19) | .093 | .09  (-0.00, 0.19) | .094 | 0.09  (-0.00, 0.19) | .093 | 0.11  (0.02, 0.20) | .045 | 0.12  (0.03, 0.20) | .033 | 0.11  (0.03, 0.20) | .035 | -0.11  (0.03, 0.20) | .111 | 0.19  (0.05, 0.33) | .120 |
| Duration follow-up |  |  | -.00  (-0.07, 0.07) | .987 | -0.00  (-0.07, 0.07) | .971 | -0.06  (-0.16, 0.03) | .212 | -0.02  (-0.16, 0.11) | .740 | -0.03  (-0.29, 0.22) | .815 | -0.03  (-0.29, 0.22) | .425 | 0.26  (-0.03, 0.56) | .082 |
| Addiction type (tobacco vs. alcohol) |  |  |  |  | 0.02  (-0.04, 0.10) | .454 | 0.10  (-0.00, 0.20) | .061 | 0.06  (-0.06, 0.19) | .299 | 0.07  (-0.18, 0.34) | .573 | 0.17  (-0.18, 0.34) | .356 | -0.04  (-0.31, 0.23) | .757 |
| Type of CBM training (ApBM vs. AtBM) |  |  |  |  |  |  | -0.10  (-0.23, 0.01) | .100 | -0.05  (-0.23, 0.11) | .493 | -0.06  (-0.27, 0.14) | .551 | -0.06  (-0.27, 0.14) | .325 | 0.18  (-0.0, 0.42) | .144 |
| Addiction type * Type of CBM training |  |  |  |  |  |  |  |  | 0.04 (-0.06, 0.16) | .401 | 0.04  (-0.13, 0.22) | .633 | 0.04  (-0.13, 0.22) | .727 | 0.20  (0.01, 0.40) | .043 |
| No. Training trials |  |  |  |  |  |  |  |  |  |  | 0.00  (-0.20, 0.21) | .950 | 0.01  (-0.20, 0.21) | .584 | -0.10  (-0.32, 0.11) | .333 |
| Condition * No. Training trials |  |  |  |  |  |  |  |  |  |  |  |  | -0.10  (-0.25, 0.05) | .293 | -0.10  (-0.23, 0.03) | .286 |
| Severity substance use |  |  |  |  |  |  |  |  |  |  |  |  |  |  | 0.21  (0.11, 0.31) | <.001 |
| **Random components** |  |  |  |  |  |  |  |  |  |  |  |  |  |  |  |  |
| $\sigma^{2}$ | 1.021 | | 1.024 | | 1.025 | | 1.025 | | 1.026 | | 1.026 | | 1.019 | | 0.998 | |
| $\tau_{00, Study}$ | 0.002 | | 0.004 | | 0.003 | | 0.002 | | 0.007 | | 0.000 | | 0.000 | | 0.000 | |
| $\tau_{11, Study}$ | 0.004 | | 0.010 | | 0.009 | | 0.007 | | 0.008 | | 0.008 | | 0.000 | | 0.003 | |
| $\rho_{01}$ | 1.000 | | 1.000 | | 1.000 | | 0.007 | | 1.000 | | 1.000 | | 1.000 | | 1.000 | |
| N_Comparisons_ | 7 | | 7 | | 7 | | 7 | | 7 | | 7 | | 7 | | 7 | |
| ICC_Study_ | 0.000 | | 0.000 | | 0.000 | | 0.000 | | 0.000 | | 0.000 | | 0.000 | | 0.000 | |
| Observations | 768 | | 768 | | 768 | | 768 | | 768 | | 768 | | 768 | | 760 | |
| ${R^{2}}/{\Omega_{0}^{2}}$ | .027/.027 | | .027/.027 | | .026/.026 | | .027/.026 | | .026/.026 | | .026/.026 | | .030/.030 | | .049/.049 | |
| AIC | 2214 | | 2234 | | 2241 | | 2244 | | 2249 | | 2266 | | 2284 | | 2186 | |

B***:*** standardized beta coefficient; *95% C.I.:* 95% confidence interval for the standardized coefficient; ***p:*** p-value; $\sigma^{2}$: intra-study variance; $\tau_{00, Study}$: random intercept variance at the study level; $\tau_{11, Study}$: random slopes variance of condition at the study level; $\rho_{01}$: correlation between $\tau_{00, Study}$ and $\tau_{11, Study}$**;** *N_Comparisons_*: number of comparisons per model; *ICC_Study_*: intra-class correlation for studies; *Observations*: number of observation in each model; ${R^{2}}/{\Omega_{0}^{2}}$: R squared / Omega squared values for each model; *AIC*: Akaike Information Criterion.

**Relapse rate.** Table S9 presents the main findings of the IPD meta-analysis on relapse. Again, AIC and ${\Delta AIC}_{Mi}$showed that model $M_{7}$ best fitted to the data (AIC = 1,489, ${\Delta AIC}_{M7-M7}$= 0) although there was no significant effect of training condition on relapse rate (OR = 0.90; *p* = .637). However, a significant effect of training condition was found in those models that did not include the number of completed training trials as a moderator (i.e., models $M_{0}$, $M_{1}{, M}_{2}$, $M_{3}$and $M_{4}$), all indicating around 16% lower chance to relapse in the training group. Although the introduction of amount of completed training trials as a moderator did not impact substantially on the chance of relapse (all ORs = 1.00; *p*s < .01), suggesting that the moderator may be discarded, models $M_{0}$ to $M_{4}$ still showed poor fit to the data compared to model $M_{7}$ (all $\Delta AIC$s > 10).

Note that model $M_{7}$ suffered from the exclusion of one comparison (Schoenmakers et al., 2010), which prevented the estimation of the parameter for the interaction effect between addiction type and type of CBM training and, similarly to the cognitive bias outcome, makes a direct comparison with the other models questionable. When examining the goodness of fit of the models including all comparisons, model $M_{5}$ was the best fitting the data (AIC = 1,558) and the most parsimonious, yet including a non-significant effect of training condition on relapse rate (OR = 1.02; *p* = .88) but a significant main effect of both type of addiction and CBM training on the relapse outcome. Tobacco use disorder showed a 245% higher chance of relapse at follow-up relative to alcohol use disorder (OR = 3.45, *p* < .001), while using ApBM training seems to have a greater positive impact on relapse than AtBM training (88% lower chance of relapse; OR = 1.88, *p* = .037).

Table S9. Hierarchical mixed-effects model results on relapse rate for the frequentist 1-stage IPD meta-analysis (95% confidence intervals between brackets)

|  | $\boldsymbol{M}_{\boldsymbol{0}}$ | | $\boldsymbol{M}_{\boldsymbol{1}}$ | | $\boldsymbol{M}_{\boldsymbol{2}}$ | | $\boldsymbol{M}_{\boldsymbol{3}}$ | | $\boldsymbol{M}_{\boldsymbol{4}}$ | | $\boldsymbol{M}_{\boldsymbol{5}}$ | | $\boldsymbol{M}_{\boldsymbol{6}}$ | | $\boldsymbol{M}_{\boldsymbol{7}}$ | |
| --- | --- | --- | --- | --- | --- | --- | --- | --- | --- | --- | --- | --- | --- | --- | --- | --- |
|  | **OR** | **p** | **OR** | **p** | **OR** | **p** | **OR** | **p** | **OR** | **p** | **OR** | **p** | **OR** | **p** | **OR** | **p** |
| **Fixed effects** |  |  |  |  |  |  |  |  |  |  |  |  |  |  |  |  |
| Intercept | 2.34  (1.01, 5.40) | .047 | 4.57  (0.89, 23.46) | .068 | 1.67  (0.66, 4.24) | .269 | 2.66  (1.17, 6.02) | .019 | 2.47  (1.08, 5.68) | .033 | 4.68  (1.49, 14.71) | .008 | 4.66  (1.46, 14.83) | .009 | 2.10  (0.45, 9.74) | .349 |
| Condition (training vs. control) | 0.84  (0.73, 0.95) | .011 | 0.84  (0.74, 0.96) | .01 | 0.85  (0.74, 0.97) | .019 | 0.84  (0.75,  0.95) | .008 | 0.84  (0.74, 0.96) | .011 | 1.02  (0.81, 1.29) | .887 | 0.92  (0.62, 1.37) | .686 | 0.90  (0.59, 1.38) | .637 |
| Duration follow-up |  |  | 0.97  (0.93, 1.02) | .350 | 1.01  (0.98,  1.04) | .477 | 1.00  (0.97, 1.02) | .752 | 1.00  (0.98, 1.03) | .768 | 1.01  (0.97, 1.04) | .719 | 1.01  (0.97, 1.05) | .647 | 1.01  (0.97, 1.05) | .636 |
| Addiction type (tobacco vs. alcohol) |  |  |  |  | 2.72  (1.74, 4.26) | <.001 | 3.92  (2.60, 5.93) | <.001 | 4.34  (2.67, 7.15) | <.001 | 3.45  (1.95, 6.12) | <.001 | 3.44  (1.93, 6.16) | <.001 | 7.05  (2.25, 22.10) | <.001 |
| Type of CBM training (ApBM vs. AtBM) |  |  |  |  |  |  | 2.03  (1.26, 3.27) | .003 | 2.04  (1.24, 3.35) | .005 | 1.88  (1.08, 3.27) | .025 | 1.86  (1.06, 3.28) | .030 | 3.24  (1.24, 8.46) | .016 |
| Addiction type * Type of CBM training |  |  |  |  |  |  |  |  | 1.28  (0.72, 2.30) | .400 | 1.61  (0.78, 3.25) | .181 | 1.67  (0.82, 3.43) | .155 | – | – |
| No. Training trials |  |  |  |  |  |  |  |  |  |  | 1.00  (1.00, 1.00) | .004 | 1.00  (1.00, 1.00) | .016 | 1.00  (1.00, 1.00) | .036 |
| Condition * No. Training trials |  |  |  |  |  |  |  |  |  |  |  |  | 1.00  (1.00, 1.00) | .54 | 1.00  (1.00, 1.00) | .538 |
| Severity substance use |  |  |  |  |  |  |  |  |  |  |  |  |  |  | 1.01  (1.00, 1.03) | .034 |
| **Random components** |  |  |  |  |  |  |  |  |  |  |  |  |  |  |  |  |
| $\tau_{00, Study}$ | 1.360 | | 1.225 | | 0.252 | | 0.088 | | 0.081 | | 0.024 | | 0.049 | | 0.088 | |
| $\tau_{11, Study}$ | 0.001 | | 0.001 | | 0.003 | | 0.000 | | 0.000 | | 0.041 | | 0.049 | | 0.054 | |
| $\rho_{01}$ | -1.000 | | -1.000 | | -1.000 | | -1.000 | | -1.000 | | -1.000 | | -1.000 | | -1.000 | |
| N_Comparisons_ | 8 | | 8 | | 8 | | 8 | | 8 | | 8 | | 8 | | 7^a^ | |
| ICC_Study_ | 0.000 | | 0.000 | | 0.000 | | 0.000 | | 0.000 | | 0.050 | | 0.049 | | 0.049 | |
| Observations | 1411 | | 1411 | | 1411 | | 1411 | | 1411 | | 1411 | | 1411 | | 1357 | |
| Deviance | 1565 | | 1564 | | 1555 | | 1546 | | 1546 | | 1536 | | 1536 | | 1465 | |
| AIC | 1577 | | 1578 | | 1571 | | 1564 | | 1566 | | 1558 | | 1560 | | 1489 | |

^a^ One comparison could not be included in model $M_{7}$ (Schoenmakers et al., 2010) due to the absence of IPD data on severity of substance use. This also affected the estimation of the parameter for the interaction effect of addiction type * type of CBM training, which could not be computed due to the presence of zero observations in one of the 2 (addiction type: alcohol or tobacco) x 2 (type of CBM training: AtBM or ApBM) levels.

OR: odds ratio for the increase in probability to relapse in the control condition compared to the training condition; *95% C.I.:* 95% confidence interval for the odds ratio; *p:* *p*-value;$\tau_{00, Study}$: random intercept variance at the study level; $\tau_{11, Study}$: random slopes variance of condition at the study level; $\rho_{01}$: correlation between $\tau_{00, Study}$ and $\tau_{11, Study}$; *N_Comparisons_*: number of comparisons per model; *ICC_Study_*: intra-class correlation for studies; *Observations*: number of observation in each model; *Deviance*: Residual Deviance; *AIC*: Akaike Information Criterion

**References**

Barr, DJ, Levy R., Scheepers C., & Tily HJ. (2013) Random effects structure for confirmatory hypothesis testing: Keep it maximal. Journal of Memory and Language, 68, 255–278.

Burnham, K. P., Anderson, D. R., & Huyvaert, K. P. (2011). AIC model selection and multimodel inference in behavioral ecology: Some background, observations, and comparisons. Behavioral Ecology and Sociobiology, 65(1), 23–35.

Burnham, K. P., & Anderson, D. R. (2002). Model Selection and Multimodel Inference: A Practical Information-Theoretic Approach (2nd ed). Ecological Modelling (Vol. 172). <http://doi.org/10.1016/j.ecolmodel.2003.11.004>

R Core Team (2017). R: A language and environment for statistical computing. R Foundation for Statistical Computing, Vienna, Austria. URL .

Table S10. Evaluation of Risk of bias in the included studies according to the criteria of the Cochrane’s Collaboration Tool

| **Study** | **Criterion** | **Judgment** | **Support for judgement** |
| --- | --- | --- | --- |
| Begh et al., 2015 | Random sequence generation (selection bias) | Low | Quote: *The trial statistician produced the sequence that allocated participants 1:1 to either attentional retraining or placebo training, using a computer-generated simple randomisation scheme ordered in random permuted blocks of four.*  Use of a computerized system to generate randomization sequence. |
|  | Allocation concealment (selection bias) | Low | Quote from study protocol (Begh et al., 2013): *The sequence was generated by the trial statistician and entered on to a dedicated online trial database by an independent programmer in the Primary Care Clinical Research and Trials Unit (PCCRTU) at the University of Birmingham. At 1 week prior to quit day, at the start of the clinic session, the therapist will access the randomisation section of the trial database and click on a button that reveals a letter (‘A’ or ‘B’) to reveal the training task to which the participant is allocated. The training tasks are contained within two folders labelled ‘Training A’ or ‘Training B’ on the study laptop, which conceals whether the procedure is AR or PT. These folders were labelled by an independent researcher prior to the start of the trial. Thus the participants, therapists and study staff will be blinded to allocation, to minimize the risk of bias*  Note: the study protocol (Begh et al., 2013) includes a questionnaire on knowledge of group allocation, which is not reported in the final report (Begh et al., 2015). However, due to the additive nature of the training program (on top of an intensive smoking cessation program with both a pharmacological and behavioral intervention), it is unlikely that participants’ awareness of their assigned training condition would have substantially affected the results and/or compliance to treatment. |
|  | Blinding of participants and personnel (performance bias) | Low | Quote from study protocol (Begh et al., 2013): *At 1 week prior to quit day, at the start of the clinic session, the therapist will access the randomisation section of the trial database and click on a button that reveals a letter (‘A’ or ‘B’) to reveal the training task to which the participant is allocated. The training tasks are contained within two folders labelled ‘Training A’ or ‘Training B’ on the study laptop, which conceals whether the procedure is AR or PT. These folders were labelled by an independent researcher prior to the start of the trial. Thus the participants, therapists and study staff will be blinded to allocation, to minimize the risk of bias.*  Blinding of study personnel and participants implemented.  Note: The study protocol (Begh et al., 2013) includes a questionnaire on knowledge of group allocation, which is not reported in the final report (Begh et al., 2015). However, due to the additive nature of the training program (on top of an intensive smoking cessation program with both a pharmacological and behavioral intervention), it is unlikely that participants’ guessing of their assigned training condition would have substantially affected the results and/or compliance to treatment. |
|  | Blinding of outcome assessment (detection bias) (cognitive bias outcomes) | Low | Cognitive bias assessed with a reaction time computerized task and administered by the clinic personnel (i.e., research nurses and stop smoking advisors), who were fully blinded to the allocated condition and trained on the delivery of the tasks by the chief investigator (Begh et al., 2013). |
|  | Blinding of outcome assessment (detection bias) (behavioral outcomes, such as drinking or smoking outcomes) | Low | Objective measure of abstinence (i.e., exhaled carbon monoxide in breath) was taken at the start of each study session by the clinic personnel (i.e., research nurses and stop smoking advisors), who was fully blinded to the allocated condition. |
|  | Incomplete outcome data addressed (attrition bias)  (short term: cognitive bias outcomes) | Low | Quote: *The analysis was carried out on all data collected initially and then with imputation for missing RT and questionnaire data but gave nearly identical results and is not reported.* |
|  | Incomplete outcome data addressed (attrition bias)  (long term: behavioral outcomes, such as drinking or smoking outcomes) | Low | Participants missed to follow-up classified as non-abstainers following reported standard guidelines.  No significant difference between groups in median number of study sessions attended. |
|  | Selective reporting (reporting bias) | Low | All primary and secondary outcomes specified in the study protocol (Begh et al., 2013) are reported in the final study report (Begh et al., 2015). |
| Clerkin et al., 2016 | Random sequence generation (selection bias) | Low | Quote: *Hence, using block randomisation (block size 12), participants were randomised into one of 4 possible conditions.*  Authors further specified in personal correspondence that the block-randomisation sequence was generated by a online randomisation. |
|  | Allocation concealment (selection bias) | Low | Authors specified in personal correspondence that conditions were labelled with numbers and only a member of the group not involved in the data collection new which conditions the numbers corresponded to. |
|  | Blinding of participants and personnel (performance bias) | Low | Quote: *Experimenters and participants were both blind with respect to participant condition.*  Personnel and participants did not know to which condition the participants’ assigned number corresponded to. Only the main PI, who was not involved in the data collection and located in another city, knew it. |
|  | Blinding of outcome assessment (detection bias) (cognitive bias outcomes) | Low | Cognitive bias assessed with a computerized reaction time task. |
|  | Blinding of outcome assessment (detection bias) (behavioral outcomes, such as drinking or smoking outcomes) | Low | Self-report measures of alcohol outcomes were administered by the experimenters, who were blinded to participants’ assigned condition. |
|  | Incomplete outcome data addressed (attrition bias)  (short term: cognitive bias outcomes) | Low | Quote: *Following Carlbring et al.’s (2012) ABM trial for SAD, the primary analyses used multilevel modeling to capture both the trajectories of symptoms within individuals (i.e., level 1) and the hypothesized between-subject moderators of these trajectories, alcohol and anxiety attention training (i.e., level 2). This method analyzes all available data for each participant, conducting intent-to-treat analyses in a manner that optimizes power for controlled clinical trials with missing data (Chakraborty & Gu, 2009).*  Use of statistical methods robust to missing data. |
|  | Incomplete outcome data addressed (attrition bias)  (long term: behavioral outcomes, such as drinking or smoking outcomes) | Low | Quote: *Following Carlbring et al.’s (2012) ABM trial for SAD, the primary analyses used multilevel modeling to capture both the trajectories of symptoms within individuals (i.e., level 1) and the hypothesized between-subject moderators of these trajectories, alcohol and anxiety attention training (i.e., level 2). This method analyzes all available data for each participant, conducting intent-to-treat analyses in a manner that optimizes power for controlled clinical trials with missing data (Chakraborty & Gu, 2009).*  Use of statistical methods robust to missing data. |
|  | Selective reporting (reporting bias) | Low | The study is registered at ClinicalTrials.gov. All pre-specified outcomes are reported in the Results. |
| Cox et al., 2015 | Random sequence generation (selection bias) | High | Quote: *After completing it, they were randomly assigned to one of the four groups, but with the constraint (a) that the four groups were approximately equivalent at baseline in mean alcohol consumption, and (b) the groups were of approximately equal size.*  Although mentioning a random allocation, the authors further specified in personal correspondence that participants were assigned to groups in sequential order (group 1, 2, 3, 4, 1, 2, 3, 4 and so on), manually counterbalancing assignment to conditions for baseline characteristics. Therefore the assignment to conditions was not based on a random sequence. |
|  | Allocation concealment (selection bias) | High | The assignment was not random and manually adjusted to counterbalance baseline characteristics. |
|  | Blinding of participants and personnel (performance bias) | Low | Quote: *A research assistant (a doctoral-level student in psychology) administered this assessment (and the subsequent assessments); she was aware of the group to which participants had been assigned. […] It would, of course, have been impossible for the person delivering each of the interventions to be unaware of the group to which participants had been assigned.*  Blinding not possible: Due to the type of intervention and study design (factorial combination of therapist-delivered AACTP and LEAP interventions), neither participants nor the experimenter could have been blinded to the assigned condition. |
|  | Blinding of outcome assessment (detection bias) (cognitive bias outcomes) | Unclear | The study did not assess this outcome. |
|  | Blinding of outcome assessment (detection bias) (behavioral outcomes, such as drinking or smoking outcomes) | High | Self-report measures of alcohol outcomes were administered by the experimenter who was aware of the assigned condition.  Likely risk of desirability bias. |
|  | Incomplete outcome data addressed (attrition bias)  (short term: cognitive bias outcomes) | Unclear | The study did not assess this outcome. |
|  | Incomplete outcome data addressed (attrition bias)  (long term: behavioral outcomes, such as drinking or smoking outcomes) | High | Quote: *Despite the high attrition rate during the posttreatment phase of the study, missing values were not replaced. We retained all participants who completed the treatment phase of the study but who dropped out at later stages. We treated the corresponding data points as missing values in the analyses.*  *We thus considered several variables on which the completers and noncompleters might have differed at baseline: RTC, SIP, SCQ, PA, NA, SWL, and LDQ. We used independent- samples t-tests to compare the completers and noncompleters on these variables (see Table 3).*  Although differences between study completers and non-completers were included in the main outcome ANCOVA analyses, no ITT analyses were carried out, excluding participants who dropped out during the treatment phase (20% of randomized participants). |
|  | Selective reporting (reporting bias) | Low | All outcomes included in the Methods are reported in the Results. However, no study protocol is available.  Authors confirmed in personal correspondence that all outcomes were reported. |
| Eberl et al., 2013  (no reply yet) | Random sequence generation (selection bias) | Unclear | Quote: *After the pretest, patients were randomly assigned to training conditions (training vs. no training).*  Although a form of randomisation was mentioned, there is insufficient information about how the randomisation sequence was generated. |
|  | Allocation concealment (selection bias) | Unclear | There is no information about how the randomisation sequence was implemented and concealed |
|  | Blinding of participants and personnel (performance bias) | Unclear | There is no information about blinding.  However blinding was probably not possible: Due to the study design (TAU + training vs. TAU only), neither participants nor the experimenters could have been blinded to the assigned condition. However, due to the already intensive nature of TAU and the study design it is unlikely that a lack of blinding would have biased the outcomes and/or compliance to the intervention (TAU therapists were independent from the study; cognitive bias outcomes assessed in computerized format, and abstinence outcome assessed one year later). |
|  | Blinding of outcome assessment (detection bias) (cognitive bias outcomes) | Low | Cognitive bias assessed with a computerized reaction time task. |
|  | Blinding of outcome assessment (detection bias) (behavioral outcomes, such as drinking or smoking outcomes) | Low | Quote: *One year after discharge, patients received a standard follow-up questionnaire asking about alcohol consumption since treatment. Participants who did not return the questionnaire were reminded by post twice and finally called by phone. In some cases (e.g., death) information was retrieved from relatives or physicians.*  One-year self-reported abstinence as part of the clinic follow-up routine, unrelated to the study experimenters. No likely risk of bias. |
|  | Incomplete outcome data addressed (attrition bias)  (short term: cognitive bias outcomes) | High | Only participants with complete correct data were analyzed (similar numbers of excluded participants per group at baseline, but almost double amount of excluded participants in the control group at post-test, with no test of between-group differences in the amount of excluded participants, nor sensitivity analyses including the excluded participants).  Post-assessment analytical sample size of A-AAT as computed from degrees of freedom in ANOVA analyses (n = 352) does not match the post-assessment A-AAT analytical sample mentioned in the Methods section (n = 341). |
|  | Incomplete outcome data addressed (attrition bias)  (long term: behavioral outcomes, such as drinking or smoking outcomes) | Low | Participants missed to follow-up classified as non-abstainers following reported standard guidelines. Results of per-protocol and ITT analyses reported.  A small incongruence in reporting exclusion data: of 509 screened patients, 11 did not fulfill inclusion criteria, 13 patients were excluded due to technical issues, and 11 patients dropped out at/after pre-test (unclear if before or after randomization). Randomized patients should be 474, not 475. However, raw data includes 475 participants. |
|  | Selective reporting (reporting bias) | High | All outcomes mentioned in the Methods section are reported in the Results.  However, no study protocol is available and the raw data file includes additional measures not mentioned not reported in the final report. |
| Elfeddali et al. 2016 | Random sequence generation (selection bias) | Low | Quote: *The respondents were randomized into one of the two conditions by means of a computerized randomization mode after entering the VPT.*  Use of a computerized system to generate randomization sequence. |
|  | Allocation concealment (selection bias) | Low | Although no information is provided on allocation concealment and on who created the computerized randomization system, condition allocation seems to be fully automatized in the online program making it very unlikely to predict allocations to conditions. |
|  | Blinding of participants and personnel (performance bias) | Low | Condition allocation was automatized in the online program and likely unknown to the experimenters. Authors further specified in personal correspondence that participants were not told to which condition they were assigned, they were told that they had a chance to be assigned to a control condition, which involved a sham training with the same number of trials to prevent that participants would know that they only do pre and post measurements. |
|  | Blinding of outcome assessment (detection bias) (cognitive bias outcomes) | Low | Cognitive bias assessed online with a computerized reaction time task. |
|  | Blinding of outcome assessment (detection bias) (behavioral outcomes, such as drinking or smoking outcomes) | Low | Self-reported smoking abstinence assessed via an online form. |
|  | Incomplete outcome data addressed (attrition bias)  (short term: cognitive bias outcomes) | High | Analysis of post-assessment completers only.  No information on significant between-group differences in post-assessment drop-out rate. |
|  | Incomplete outcome data addressed (attrition bias)  (long term: behavioral outcomes, such as drinking or smoking outcomes) | Low | Participants missed to follow-up classified as non-abstainers as similarly done in the other studies. Results of abstinence rate per-protocol and ITT analyses reported.  No significant difference in 6-month drop-out rate between the two conditions. |
|  | Selective reporting (reporting bias) | Low | The study is registered in the Dutch Trial Registry. All primary outcomes are reported as pre-specified in the trial registration.  Although risk of reporting bias is likely low, an extra 12-months follow-up was included in the trial record but not mentioned in the final report, as well as secondary outcomes on how the participants experienced participation to the study. |
| Kong et al., 2015 | Random sequence generation (selection bias) | Low | Quote: *Of the 75 adolescents presented to participate in the study, 60 (80%) were randomized to receive weekly CBT with either weekly CBM (n = 29) or with sham training (n = 31) (See Figure 1 for CONSORT diagram).*  Authors further specified in personal correspondence that the randomisation sequence was automatically generated in the computerised software used to deliver the training. |
|  | Allocation concealment (selection bias) | Low | Authors specified in personal correspondence that the randomisation sequence was automated in the computerised program, therefore it was unpredictable to which condition participants were assigned to. Further, a research assistant kept trace of the randomized condition and assigned unique IDs to all randomized participants. This assignment of IDs was kept in a password protected document. Staff involved in the study did not have access to this document. |
|  | Blinding of participants and personnel (performance bias) | Low | Quote: *All therapists were blinded to the CBM and sham condition.*  Therapists involved in the study were blind to participants’ condition allocation.  No explicit information is provided about participants blinding, although given the intervention and study design (TAU + CBM vs TAU + sham training), it is likely that participants were also blind to their condition. |
|  | Blinding of outcome assessment (detection bias)  (cognitive bias outcomes) | Low | Cognitive bias assessed with a computerized reaction time task. |
|  | Blinding of outcome assessment (detection bias)  (behavioral outcomes, such as drinking or smoking outcomes) | Low | Seven-day self-reported abstinence is biochemically verified using cotinine levels.  Although there is no information on who carried out the outcome assessments and how, the objective verification of the outcome assessment indicates a low risk of bias. |
|  | Incomplete outcome data addressed (attrition bias)  (short term: cognitive bias outcomes) | Low | Although the analysis included follow-up completers only, there was no significant difference in follow-up drop-out rate between conditions. |
|  | Incomplete outcome data addressed (attrition bias)  (long term: behavioral outcomes, such as drinking or smoking outcomes) | Low | Participants missed to follow-up classified as non-abstainers as similarly done in the other studies.  Results of abstinence rate analysis are reported per ITT. |
|  | Selective reporting (reporting bias) | Low | All outcomes mentioned in the Methods section are reported in the Results.  Authors confirmed in personal correspondence that all outcomes were reported. |
| Lopes et al., 2014 | Random sequence generation (selection bias) | High | Quote: *Participants were randomly allocated to one of three conditions defined according to the number of sessions of ABM*  Authors further specified in personal correspondence that participants were assigned to condition in sequential order (group 1, 2, 3, 1,2,3, etc.) once the arrived at the lab for the baseline assessment. Although participants randomly arrived in the lab, the randomisation sequence itself did not include a fully random component and it was predictable to which condition the next participant would have been assigned to. |
|  | Allocation concealment (selection bias) | High | The allocation to condition was fully predictable and not concealed during the experimental procedure. |
|  | Blinding of participants and personnel (performance bias) | Low | Authors specified in personal correspondence that participants were not informed about the main differences between conditions, therapists involved in the study did not know to which group patients were assigned to. The researchers collecting the data were aware to which condition participants were assigned to, but both cognitive bias and smoking abstinence were assessed with measures not at risk of being influenced by assessors’ judgment. |
|  | Blinding of outcome assessment (detection bias)  (cognitive bias outcomes) | Low | Cognitive bias assessed with a computerized reaction time task. |
|  | Blinding of outcome assessment (detection bias)  (behavioral outcomes, such as drinking or smoking outcomes) | Low | Objective measure of abstinence (i.e., exhaled carbon monoxide in breath) taken at each assessment. |
|  | Incomplete outcome data addressed (attrition bias)  (short term: cognitive bias outcomes) | Low | No drop-outs at the 24-hour post-assessment, which is the time point included in the meta-analysis.  In the remaining follow-ups drop-outs were treated as missing data, with no information on significant between-group differences in drop-out rate. |
|  | Incomplete outcome data addressed (attrition bias)  (long term: behavioral outcomes, such as drinking or smoking outcomes) | High | Participants missed to follow-up treated as missing data. Per-protocol analysis of abstinence outcome. |
|  | Selective reporting (reporting bias) | Low | All outcomes mentioned in the Methods section are reported in the Results. However, no study protocol is available.  Authors confirmed in personal correspondence that all outcomes are reported. |
| Machulska et al., 2016 | Random sequence generation (selection bias) | Low | Quote: *Afterward, smokers were randomly assigned either to the experimental or to the sham-control condition.*  Authors further specified in personal correspondence that the randomisation sequence was generated with a statistical software. |
|  | Allocation concealment (selection bias) | High | Authors specified in personal correspondence that no concealment strategy was used. The random allocation schedule was open and accessible to the main experimenter. |
|  | Blinding of participants and personnel (performance bias) | High | Authors specified in personal correspondence that participants were blind to which condition they were assigned to and were given the same information in both conditions. However, the study personnel were not blind to condition assignment. |
|  | Blinding of outcome assessment (detection bias)  (cognitive bias outcomes) | Low | Cognitive bias assessed with a computerized reaction time task. |
|  | Blinding of outcome assessment (detection bias)  (behavioral outcomes, such as drinking or smoking outcomes) | Low | Self-reported amount of cigarettes smoked assessed by phone or email. Although it is unclear if and how the experimenters were blinded to participants’ assigned condition, the assessment method does not pose a serious risk of bias in favor of one or the other condition. |
|  | Incomplete outcome data addressed (attrition bias)  (short term: cognitive bias outcomes) | Low | Quote: *To fill in missing data, we used modified Intention-To-Treat (ITT) principles (Fergusson et al., 2002) and the Last-Observation- Carried-Forward (LOCF) method.*  Also, although no attrition data is reported, there is no significant difference in amount of completed study sessions between the two groups. |
|  | Incomplete outcome data addressed (attrition bias)  (long term: behavioral outcomes, such as drinking or smoking outcomes) | High | Quote: *To fill in missing data, we used modified Intention-To-Treat (ITT) principles (Fergusson et al., 2002) and the Last-Observation- Carried-Forward (LOCF) method.*  Although single imputation of missing data was carried out, no attrition data is reported. However, there is no significant difference in amount of completed study sessions between the two groups.  Six participants were excluded due to excessive error rate in the cognitive bias task. No sensitivity analyses were carried out for the behavioral outcomes including these 6 participants.  Although not formally posing a risk of bias, note that the original sample comprised extra 41 participants who withdrew during or immediately after the first session (22% of the included participants), who could not be included in ITT analyses. |
|  | Selective reporting (reporting bias) | Low | All outcomes mentioned in the Methods section are reported in the Results. However, no study protocol is available.  Authors confirmed in personal correspondence that all outcomes are reported. Additional genetic outcomes are currently under analysis, although not relevant for this study. |
| McHugh et al., 2010 | Random sequence generation (selection bias) | Low | Quote: *Participants were randomized to AT or control training (CT) conditions and first completed the study questionnaire packet followed by stress provocation and cue exposures (the order of which was established through randomization).*  The authors further specified in personal correspondence that a random number generator was used and that randomisation was stratified by gender. |
|  | Allocation concealment (selection bias) | Low | The authors specified in personal correspondence that the randomisation sequence was concealed with a numerical code created by a lab member not involved in the study. Study personnel received the numerical code but did not know what condition the code corresponded to until this was unblinded at the completion of the study. |
|  | Blinding of participants and personnel (performance bias) | Low | The authors specified in personal correspondence that participants were not informed of their randomization status and a credible control was used. Study staff had access to the numerical code to identify which task to administer, but did not know what condition the code corresponded to until this was unblinded at the completion of the study (the pi was also not aware, a lab member not involved in the study assigned the numerical code). |
|  | Blinding of outcome assessment (detection bias)  (cognitive bias outcomes) | Low | Cognitive bias assessed with a computerized reaction time task. |
|  | Blinding of outcome assessment (detection bias)  (behavioral outcomes, such as drinking or smoking outcomes) | Unclear | The study did not assess this outcome |
|  | Incomplete outcome data addressed (attrition bias)  (short term: cognitive bias outcomes) | High | One-session study, no missing data in cognitive bias outcome.  In the analyses, despite a similar amount of participants was excluded in the two groups, exclusion was based on an extremely conservative criterion based on task performance (only very accurate and fast responding participants included in the analysis), with no sensitivity analyses including the excluded participants (n = 12). |
|  | Incomplete outcome data addressed (attrition bias)  (long term: behavioral outcomes, such as drinking or smoking outcomes) | Unclear | The study did not assess this outcome |
|  | Selective reporting (reporting bias) | Low | All outcomes mentioned in the Methods section are reported in the Results. However, no study protocol is available.  Authors confirmed in personal correspondence that all specified outcomes were reported. |
| Schoenmakers et al., 2010 | Random sequence generation (selection bias) | Low | Quote: *Participants were randomly assigned to one of the two experimental groups: the ABM group and a control group, stratified by gender and treatment center. A randomization sequence that was generated by http://www.randomization.com was used for each stratum.*  Use of a computerized system to generate a randomization sequence. |
|  | Allocation concealment (selection bias) | High | *Quote: Only the experimenter had access to this sequence.*  The main experimenter had access to the randomization sequence. |
|  | Blinding of participants and personnel (performance bias) | Low | Quote: *Therapists and patients were not informed about which group patients had been assigned to.*  Patients were also given the same, generic information about the two conditions: *The researchers explained to the patients that their addiction is partly maintained by an uncontrolled attention for alcohol-related objects, and that this training pro- gram would test the effectiveness of two interventions to increase control over their attention for alcohol. Patients in both training groups were given the same information to prevent suspicion about being assigned to a non-training control group*  Incomplete blinding: the main experimenter was aware of the assigned condition. However, this is not likely to pose a risk of bias. |
|  | Blinding of outcome assessment (detection bias)  (cognitive bias outcomes) | Low | Cognitive bias assessed with a computerized reaction time task. |
|  | Blinding of outcome assessment (detection bias)  (behavioral outcomes, such as drinking or smoking outcomes) | Low | Quote: *Three months after the last session, patients who had completed the intervention were sent a follow-up questionnaire about their treatment status and alcohol use in the past 3 months. Additionally, medical files of patients were consulted for the same variables.*  *I*ntegration of self-reported alcohol use with patient’s medical records. |
|  | Incomplete outcome data addressed (attrition bias)  (short term: cognitive bias outcomes) | Low | Similar drop-out rate between the two conditions (n = 18 and n = 19).  Note that ITT analyses were conducted, although using a controversial simple imputation method (i.e., last observation carried forward). |
|  | Incomplete outcome data addressed (attrition bias)  (long term: behavioral outcomes, such as drinking or smoking outcomes) | Unclear | Underpowered sample for abstinence rate analysis, outcome described (only completers) but not analyzed. |
|  | Selective reporting (reporting bias) | High | All outcomes mentioned in the Methods section are reported in the Results. However, no study protocol is available.  Authors reported in personal correspondence that some secondary outcomes were not reported in the final paper. |
| C. E. Wiers et al., 2015 | Random sequence generation (selection bias) | High | Quote (Wiers CE et al., 2015a): *Patients were randomly assigned to a CBM or a placebo group. Patients were recruited within the first week of clinic entrance, and per week a maximum of four patients were included to the study. Because of practical reasons of training, all participants in one week were selected to be part of one training method (either training type ‘1’ or ‘2’) with one response type (either push landscape/pull portrait or pull landscape/push portrait pictures). There was no selection bias in time of clinic entrance. […] In the last weeks of the study, patients were assigned to groups while taking into consideration their age, years of education and drinking behavior, to aim for matched groups for these variables.*  Quote (Wiers CE et al., 2015b): *Patients were randomly assigned to receive bias modification training or sham training.*  The authors further specified in personal correspondence that participants were assigned to groups in blocks of three in alternating order. Although participants randomly enrolled in the study, the randomisation sequence itself did not include a fully random component and it was predictable to which condition the next participant would have been assigned to. |
|  | Allocation concealment (selection bias) | Low | Quote (Wiers CE et al., 2015a: *Both the experimenter and the trainers were always blind to whether training 1 or 2 was CBM or placebo. This information was written on a sheet, which was open to the experimenter only after data collection*  The two randomised conditions were labelled with numbers and revealed only upon completion of data collection. |
|  | Blinding of participants and personnel (performance bias) | Low | Quote (Wiers CE et al., 2015a): *In a double-blind placebo-controlled design, patients were randomly assigned to a CBM training group or a placebo training group and performed the respective training for three weeks. […]*  *Both the experimenter and the trainers were always blind to whether training 1 or 2 was CBM or placebo. This information was written on a sheet, which was open to the experimenter only after data collection.*  Double blinding of study personnel ensured. Due to the additive nature of the training program (on top of a 3-month intensive inpatient CBT program), it is unlikely that participants’ awareness of their assigned training condition would have substantially affected the results and/or compliance to treatment. |
|  | Blinding of outcome assessment (detection bias)  (cognitive bias outcomes) | Low | Cognitive bias assessed with a computerized reaction time task. |
|  | Blinding of outcome assessment (detection bias)  (behavioral outcomes, such as drinking or smoking outcomes) | Low | Although the study did not address this outcome, one-year abstinence data was included in the raw data set.  The authors informed through personal correspondence that one-year abstinence was assessed with a self-report questionnaire sent to patients as part of the clinic follow-up routine, unrelated to the study experimenters, suggesting no likely risk of bias. |
|  | Incomplete outcome data addressed (attrition bias)  (short term: cognitive bias outcomes) | Low | The study was designed to test brain activity associated with training, hence missing data in the cognitive bias outcome refer to exclusion of participants for whom it was not possible to run the target analyses. |
|  | Incomplete outcome data addressed (attrition bias)  (long term: behavioral outcomes, such as drinking or smoking outcomes) | Low | Although one-year abstinence rate is included in the raw data, the study did not address this outcome.  The authors informed through personal correspondence that no responders were contacted multiple times. In case of further no response, they were considered as relapsing, similarly to the criteria used in other studies. |
|  | Selective reporting (reporting bias) | High | All outcomes mentioned in the Methods section are reported in the Results. However, no study protocol is available and the raw data file includes unreported abstinence data. The authors further specified that this and other measures were included in the study protocol and have been later published separately. |
| Wiers et al., 2011 | Random sequence generation (selection bias) | High | Quote: *Patients were assigned randomly to one of four conditions.*  The authors further specified in personal correspondence that participants were assigned to groups in sequential order (group 1, 2, 3, 4, 1, 2, 3, 4 and so on). Although participants randomly enrolled in the study, the randomisation sequence itself did not include a fully random component and it was predictable to which condition the next participant would have been assigned to. |
|  | Allocation concealment (selection bias) | High | The assignment was not at random; therefore the assignment of participants to condition was predictable. |
|  | Blinding of participants and personnel (performance bias) | Low | Quote: *Therapists were blind to whether the training was real or sham, but they knew which group was the no-training control group.*  The additive nature of the intervention (on top of a 3-month intensive inpatient CBT program) and of the study design (two training conditions vs. two control conditions, one receiving no intervention and one receiving sham training) indicates a low risk of bias (results did not differ between the two control conditions, which were collapsed together in the main analyses). |
|  | Blinding of outcome assessment (detection bias)  (cognitive bias outcomes) | Low | Cognitive bias assessed with a computerized reaction time task. |
|  | Blinding of outcome assessment (detection bias)  (behavioral outcomes, such as drinking or smoking outcomes) | Low | Quote: *One year after treatment discharge, all patients received a follow-up questionnaire inquiring about their use of alcohol and other substances. Participants who did not return the questionnaire were reminded by mail and telephone. In cases involving death or relapse, information was obtained from patients’ relatives.*  One-year self-reported abstinence as part of the clinic follow-up routine, unrelated to the study experimenters. No likely risk of bias. |
|  | Incomplete outcome data addressed (attrition bias)  (short term: cognitive bias outcomes) | Low | Quote: *The analytical sample for the AAT was 173: 27 participants (13 in the experimental group and 14 in the control group) missed one of the two assessment AATs, and the data of another 14 participants (6 in the experimental group and 8 in the control group) had to be discarded because of excessive errors (> 35% error rate in at least one AAT; chance performance would have yielded a 50% error rate). The analytical sample for the IAT was 181: 18 participants (10 in the experimental group and 8 in the control group) missed one or two IATs, and another 15 participants (7 in the experimental group and 8 in the control group) had to be discarded because of excessive errors (> 35% error rate in at least one IAT).*  Although only completers were analyzed, amount of missing data for both cognitive bias outcomes (AAT and IAT) was similar between the two main conditions. |
|  | Incomplete outcome data addressed (attrition bias)  (long term: behavioral outcomes, such as drinking or smoking outcomes) | Low | Quote: *The 1-year follow-up data were analyzed for successfully retrieved outcomes and with ITT. Following guidelines of the German Addiction Society, we used success as a binary outcome variable. Success was defined as no relapse or a single lapse shorter than 3 days that was ended by the patient without further negative consequences. No success was defined as relapse or death, and in ITT analyses, no information and refusal were included in this category.*  Participants missed to follow-up classified as non-abstainers following reported standard guidelines. Results of per-protocol and ITT analyses reported. |
|  | Selective reporting (reporting bias) | Low | All outcomes mentioned in the Methods section are reported in the Results. However, no study protocol is available.  The authors confirmed in personal correspondence that all specified primary outcomes were reported. |
| R.W. Wiers et al., 2015 | Random sequence generation (selection bias) | Low | Quote: *The remaining 615 adults gave informed consent online and were randomly assigned to one of five conditions.*  Authors further specified in personal correspondence that the randomisation sequence was generated by a computerised system. |
|  | Allocation concealment (selection bias) | Low | Condition allocation was automatized in the online program making it very unlikely to predict allocations to conditions. |
|  | Blinding of participants and personnel (performance bias) | Low | Condition allocation was fully automatized in the online program and no personnel was involved (fully online study). Authors further specified in personal correspondence that participants were informed that they would receive one version of many training-interventions to help them curb their drinking, of which the efficacy was investigated. Participants did not know the different training-versions, therefore making it impossible for them to know which condition they were assigned to. |
|  | Blinding of outcome assessment (detection bias)  (cognitive bias outcomes) | Low | Cognitive bias assessed with a computerized reaction time task. |
|  | Blinding of outcome assessment (detection bias)  (behavioual outcomes, such as drinking or smoking outcomes) | Low | Self-reported drinking amount via an online form. The assessment method does not pose a serious risk of bias in favor of one of the conditions. |
|  | Incomplete outcome data addressed (attrition bias)  (short term: cognitive bias outcomes) | Unclear | Quote: *We focus this report on the main outcome variables, which are related to alcohol use. In addition, attentional bias and approach bias were assessed at pre- and posttest, but these data were of suboptimal quality, and reporting them would require far more space. Some additional questionnaires were also included, but they are not reported due to space limitations. The general pattern of results was the same as reported here: Beneficial changes across all conditions.*  Data on cognitive bias outcomes explicitly not reported due to space limitations. |
|  | Incomplete outcome data addressed (attrition bias)  (long term: behavioral outcomes, such as drinking or smoking outcomes) | Low | Multiple imputation used to handle missing data. Per protocol and ITT results reported. |
|  | Selective reporting (reporting bias) | High | Quote: *We focus this report on the main outcome variables, which are related to alcohol use. In addition, attentional bias and approach bias were assessed at pre- and posttest, but these data were of suboptimal quality, and reporting them would require far more space. Some additional questionnaires were also included, but they are not reported due to space limitations. The general pattern of results was the same as reported here: Beneficial changes across all conditions.*  The report explicitly mentions that additional outcomes (including cognitive bias outcomes) were not reported due to space limitations.  No study protocol is available. |
| Wittekind et al., 2015 | Random sequence generation (selection bias) | Low | Quote: *Participants who completed the baseline survey were randomly allocated to one of three conditions (standard AAT [sAAT], modified AAT [mAAT], waitlist control group) in pseudo-random order.*  Authors further specified in personal correspondence that the randomisation sequence was generated by a online randomisation. |
|  | Allocation concealment (selection bias) | High | Authors specified in personal correspondence that allocation assignment was not concealed. The primary investigator generated the sequence and also randomized participants. |
|  | Blinding of participants and personnel (performance bias) | Low | The study ran online with no personnel interacting with participants. The authors further specified in personal correspondence that participants were blinded about which training condition they received. Participants were told in the instruction that two different versions of the training were to be compared to a waitlist control group. When participants were emailed their group assignment, both the standard and the modified AAT group were informed that they received the program immediately, whereas participants in the waitlist control group were told that they would receive the training after the post-assessment. Consequently, participants were not aware of which training version they received, but were aware if they were assigned to the waitlist condition (hence, completing the training intervention later than others). Further, the modified training version was not a real placebo, but rather a modified training condition. |
|  | Blinding of outcome assessment (detection bias)  (cognitive bias outcomes) | Unclear | The study did not assess this outcome. |
|  | Blinding of outcome assessment (detection bias)  (behavioral outcomes, such as drinking or smoking outcomes) | Low | Self-reported amount of cigarettes assessed via an online form. |
|  | Incomplete outcome data addressed (attrition bias)  (short term: cognitive bias outcomes) | Unclear | The study did not assess this outcome. |
|  | Incomplete outcome data addressed (attrition bias)  (long term: behavioral outcomes, such as drinking or smoking outcomes) | Low | No significant difference in completion rate between the two conditions.  Both per-protocol and ITT analyses for cigarette reduction were reported, although using a controversial simple imputation method (i.e., last observation carried forward). |
|  | Selective reporting (reporting bias) | High | All outcomes mentioned in the Methods section are reported in the Results. However, no study protocol is available.  The authors confirmed in personal correspondence that all specified primary outcomes were reported. A secondary outcome (i.e., depressive symptoms) was also measured but not reported. |
